# Complications and adverse events in lymphadenectomy of the inguinal area: worldwide expert consensus

**DOI:** 10.1093/bjsopen/zrae056

**Published:** 2024-07-11

**Authors:** René Sotelo, Aref S Sayegh, Luis G Medina, Laura C Perez, Anibal La Riva, Michael B Eppler, José Gaona, Marcos Tobias-Machado, Philippe E Spiess, Curtis A Pettaway, Antonio Carlos Lima Pompeo, Pablo Aloisio Lima Mattos, Timothy G Wilson, Gustavo M Villoldo, Eric Chung, Aldo Samaniego, Antonio Augusto Ornellas, Vladimir Pinheiro, Eder S Brazão, David Subira-Rios, Leandro Koifman, Stênio de Cassio Zequi, Humberto M Pontillo Z, José de Ribamar Rodrigues Calixto, Rafael Campos Silva, B Mark Smithers, Simone Garzon, Oliver Haase, Antonio Sommariva, Robert Fruscio, Francisco Martins, Pedro S de Oliveira, Giovanni Battista Levi Sandri, Marco Clementi, Juan Astigueta, Islam H Metwally, Rasiah Bharathan, Tarun Jindal, Yasuhiro Nakamura, Hisham Abdel Mageed, Sakthiushadevi Jeevarajan, Ramón Rodriguez Lay, Herney Andrés García-Perdomo, Omaira Rodríguez González, Saum Ghodoussipour, Inderbir Gill, Giovanni E Cacciamani

**Affiliations:** Catherine and Joseph Aresty Department of Urology, University of Southern California Institute of Urology, Keck School of Medicine, University of Southern California, Los Angeles, California, USA; Catherine and Joseph Aresty Department of Urology, University of Southern California Institute of Urology, Keck School of Medicine, University of Southern California, Los Angeles, California, USA; Department of Surgery, MedStar Franklin Square Medical Center, Baltimore, Maryland, USA; Catherine and Joseph Aresty Department of Urology, University of Southern California Institute of Urology, Keck School of Medicine, University of Southern California, Los Angeles, California, USA; Catherine and Joseph Aresty Department of Urology, University of Southern California Institute of Urology, Keck School of Medicine, University of Southern California, Los Angeles, California, USA; Department of Surgery, Johns Hopkins University School of Medicine, Baltimore, Maryland, USA; Catherine and Joseph Aresty Department of Urology, University of Southern California Institute of Urology, Keck School of Medicine, University of Southern California, Los Angeles, California, USA; Department of General Surgery, Digestive Disease & Surgery Institute, Cleveland Clinic Foundation, Cleveland, Ohio, USA; Catherine and Joseph Aresty Department of Urology, University of Southern California Institute of Urology, Keck School of Medicine, University of Southern California, Los Angeles, California, USA; Universidad de Santander, Instituto Uromédica, Bucaramanga, Colombia; Department of Urology, Instituto do Câncer Arnaldo Vieira de Carvalho, São Paulo, Brazil; Department of Genitourinary Oncology and Tumor Biology, Moffitt Cancer Center, Tampa, Florida, USA; The University of Texas, M.D. Anderson Cancer Center, Houston, Texas, USA; Department of Urology, Faculdade de Medicina do ABC, São Paulo, Brazil; Department of Urology, Associação Piauiense de Combate ao Câncer, Teresina, Piauí, Brazil; Department of Urology, Providence St. John’s Cancer Institute, Santa Monica, California, USA; Department of Urology, Instituto Alexander Fleming, Buenos Aires, Argentina; Department of Urology, Princess Alexandra Hospital, University of Queensland, Brisbane, Queensland, Australia; Department of Urology, Servicio de Urología del Hospital Central del Instituto de Previsión Social, Asunción, Paraguay; Departamento de Urologia, Instituto Nacional do Câncer do Brasil (INCA), Rio de Janeiro, Brazil; Department of Urology, AC Camargo Cancer Center, São Paulo, Brazil; Department of Urology, AC Camargo Cancer Center, São Paulo, Brazil; Department of Urology, Gregorio Marañon Universitary Hospital, Madrid, Spain; Serviço de Urologia, Hospital Municipal Souza Aguiar, Rio de Janeiro, Rio de Janeiro, Brazil; Department of Urology, AC Camargo Cancer Center-São Paulo, São Paulo, Brazil; Department of Urology, National Institute for Science and Technology in Oncogenomics and Therapeutic Innovation, São Paulo, Brazil; Graduate School of Urology, Escola Paulista de Medicina-Universidade Federal de São Paulo, São Paulo, Brazil; Department of Surgery, Sant Jaume of Calella Hospital, Barcelona, Spain; Department of Medicine II, Federal University of Maranhão, São Luís, Massachusetts, Brazil; Department of Urology, Hospital Universitário Presidente Dutra—HUPD/UFMA, São Luís, Maranhão, Brazil; University of Queensland, Queensland Melanoma Project, Princess Alexandra Hospital, Brisbane, Queensland, Australia; Department of Surgery, Dentistry, Pediatrics, and Gynecology, University of Verona, Verona, Italy; Department of Surgery, University Medicine Berlin—Charité, Berlin, Germany; Veneto Institute of Oncology Institute Oncology Veneto, Istituto Di Ricovero e Cura a Carattere Scientifico, Padova, Italy; Department of Medicine and Surgery, University of Milan Bicocca, Azienda Socio Sanitaria Territoriale Monza, Italy; Department of Urology, Centro Hospitalar Universitário Lisboa Norte, Hospital de Santa Maria, Lisbon, Portugal; Department of Urology, Centro Hospitalar Universitário Lisboa Norte, Hospital de Santa Maria, Lisbon, Portugal; Department of Surgery, ASL Frosinone, Frosinone, Italy; Department of Medicine, Health and Life, University of L'Aquila, L'Aquila, AQ, Italy; Department of Urology, Universidad Privada Antenor Orrego, Trujillo, Perú; Surgical Oncology Department, Oncology Center Mansoura University (OCMU), Mansoura, Egypt; Department of Gynaecological Oncology, Medical University of Vienna, Vienna, Austria; Department of Uro-oncology, Narayana Super Speciality Hospital, Howrah, India; Department of Skin Oncology/Dermatology, Saitama Medical University International Medical Center, Saitama, Japan; Surgical Oncology Department, National Cancer Institute Cairo University, Cairo, Egypt; Department of Surgical Oncology, Regional Cancer Centre, Kanchipuram, Tamil Nadu, India; Clinic Urology Service, Complejo Hospitalario Metropolitano, Madrid, Panama; Division of Urology/Urooncology, Department of Surgery, School of Medicine, Universidad del Valle, Cali, Colombia; Chief of Surgical Department, Clínicas Caracas Hospital, Faculty of Medicine, Central University of Venezuela, Caracas, Venezuela; Sections of Urologic Oncology, Rutgers Cancer Institute of New Jersey and Rutgers Robert Wood Johnson Medical School, New Brunswick, New Jersey, USA; Catherine and Joseph Aresty Department of Urology, University of Southern California Institute of Urology, Keck School of Medicine, University of Southern California, Los Angeles, California, USA; Catherine and Joseph Aresty Department of Urology, University of Southern California Institute of Urology, Keck School of Medicine, University of Southern California, Los Angeles, California, USA

## Abstract

**Background:**

Inguinal lymph node dissection plays an important role in the management of melanoma, penile and vulval cancer. Inguinal lymph node dissection is associated with various intraoperative and postoperative complications with significant heterogeneity in classification and reporting. This lack of standardization challenges efforts to study and report inguinal lymph node dissection outcomes. The aim of this study was to devise a system to standardize the classification and reporting of inguinal lymph node dissection perioperative complications by creating a worldwide collaborative, the complications and adverse events in lymphadenectomy of the inguinal area (CALI) group.

**Methods:**

A modified 3-round Delphi consensus approach surveyed a worldwide group of experts in inguinal lymph node dissection for melanoma, penile and vulval cancer. The group of experts included general surgeons, urologists and oncologists (gynaecological and surgical). The survey assessed expert agreement on inguinal lymph node dissection perioperative complications. Panel interrater agreement and consistency were assessed as the overall percentage agreement and Cronbach’s α.

**Results:**

Forty-seven experienced consultants were enrolled: 26 (55.3%) urologists, 11 (23.4%) surgical oncologists, 6 (12.8%) general surgeons and 4 (8.5%) gynaecology oncologists. Based on their expertise, 31 (66%), 10 (21.3%) and 22 (46.8%) of the participants treat penile cancer, vulval cancer and melanoma using inguinal lymph node dissection respectively; 89.4% (42 of 47) agreed with the definitions and inclusion as part of the inguinal lymph node dissection intraoperative complication group, while 93.6% (44 of 47) agreed that postoperative complications should be subclassified into five macrocategories. Unanimous agreement (100%, 37 of 37) was achieved with the final standardized classification system for reporting inguinal lymph node dissection complications in melanoma, vulval cancer and penile cancer.

**Conclusion:**

The complications and adverse events in lymphadenectomy of the inguinal area classification system has been developed as a tool to standardize the assessment and reporting of complications during inguinal lymph node dissection for the treatment of melanoma, vulval and penile cancer.

## Teaser

The complications and adverse events in lymphadenectomy of the inguinal area classification system has been developed as a tool to standardize the assessment and reporting of complications during inguinal lymph node dissection for the treatment of melanoma, vulvar and penile cancer.

## Introduction

Inguinal lymph node dissection (ILND) plays an important role in the management of melanoma, penile and vulval cancer^1–5^. ILND series report a wide range of associated morbidity rates (3–97%)^1,6–9^. It is generally considered a procedure with a high risk of perioperative complications, with more than 50% of patients reporting at least one adverse event (AE)^10,11^.

ILND is associated with various types of intraoperative and postoperative complications and AEs, including skin necrosis, wound dehiscence, infection, neurovascular injury, lymphocoele, lymphorrhoea and lymphoedema^1,9,10,12–16^. However, there is significant heterogeneity in the surgical literature in terms of how ILND-associated complications are classified and reported. This lack of standardization challenges any effort to study and report ILND outcomes^1^.

In a recent systematic review, 25% of studies documented AEs after ILND with only 50% of the criteria proposed by the European Association of Urology (EAU) guidelines recommendation^1,17^. For some specific complications, such as lymphoedema, numerous classifications exist aiming to standardize the severity grading and management. Yet, the concordance between these classifications can be variable when evaluating lower extremities, underscoring the need for a unified approach to assessing outcomes post-ILND across all specialties. This standardization is crucial to enhance the quality of the data, more so considering the rarity of the conditions that are treated with ILND^1,18^.

The complications and adverse events in lymphadenectomy of the inguinal area (CALI) collaboration was established to devise a system to standardize the classification and reporting of perioperative ILND complications. Various efforts have been made in the surgical community to standardize how perioperative AEs are reported, graded and studied^17,19–27^, and the CALI project aspires to contribute to the field of ILND.

This paper reports the results of the CALI collaboration’s 3-round Delphi survey to establish a new perioperative AE and complication classification system for ILND. This classification system was developed with the input of global experts, and it can be widely utilized by the greater surgical community.

## Methods

### Study design

A modified Delphi consensus approach^28^ surveyed an international group of experts in ILND for melanoma, penile and vulval cancer diagnosis and treatment. The group of experts included general surgeons, gynaecological oncologists, surgical oncologists and urologists. The survey assessed expert agreement on perioperative complications and AEs clustered in macro- and microcategories that were established based on the results of our previously published systematic review on ILND complications^1^. The goal of this systematic review was to identify ILND complication and AE reporting to inform this newly developed classification system.

The CALI study was reviewed and approved by the institutional review board (IRB) (UP-22-00368) and is registered on clinicaltrials.gov (NCT05388786). The results of the Delphi consensus are provided according to the Checklist for Reporting Results of Internet E-Surveys (CHERRIES) and to the Accurate Consensus Reporting Document (ACCORD) guidelines (*Supplementary material methods*)^29,30^.

### Study population and survey distribution

The list of experts invited to participate in the CALI Delphi survey were corresponding/senior authors of the articles identified from a previously published systematic review^1^. A snowball method was used, asking experts that agreed to participate in the survey to identify other experts for participation^31^.

A total of 47 experienced surgeons were contacted via e-mail and enrolled in the modified Delphi consensus survey. The survey was administered from July to December 2022 using Google Forms (https://docs.google.com/forms/).

The development of new definitions was rooted in a comprehensive literature review, followed by expert consensus within the CALI group^1^. This process is detailed in the *Supplementary material*. For example, some of the definitions come from the Common Terminology Criteria for Adverse Events from the US National Cancer Institute (CTCAE) or the Center for Disease Control and Prevention (CDC), Consensus Document of the International Society of Lymphology. When there were no standardized definitions, these were formulated from expert opinion (*Supplementary material appendices*).

Members of the CALI steering committee did not participate in the Delphi survey to avoid introducing potential bias. Multiple iterations with feedback were used to achieve consensus (greater than 80% agreement).

In the first round of the Delphi survey, survey participants were asked to report their demographics and surgical expertise, including country of practice, years of practice, surgical specialty, type of malignancy treated using ILND, type of ILND surgical approach and annual case volume. During the first round, participants were asked for their level of agreement using a 1 (strongly disagree) to 5 (strongly agree) Likert scale on a series of perioperative complications and definitions for inclusion in the new classification system. Respondents were surveyed on whether perioperative complications should be classified as intraoperative and/or postoperative and subclassified into macro- and microcategories and defined according to the existing classifications and definitions^20,21,32–36^. In cases where limitations in ILND perioperative complication classification systems were identified based on the previous systematic review^1^, new definitions were provided and experts again rated their level of agreement from 1 (strongly disagree) to 5 (strongly agree). Lastly, experts were encouraged to provide written feedback in free-text form. These responses were reviewed to improve the proposed classification system in a standardized fashion.

In the second round of the Delphi survey, the experts were asked to assess the changes implemented from the first round using a 1 to 5 Likert scale. Despite reaching a consensus on most of the classification systems and definitions following the first round, the system was refined by asking experts to provide written feedback, even in instances where consensus was reached.

Lastly, in the third round of the Delphi survey, experts were asked to evaluate the changes implemented from the second round, again using the 5-point Likert scale. After reaching consensus on each item for inclusion in the CALI classification system, a representative classification system template for assessing and reporting perioperative complications and AEs associated with ILND was created.

### Statistical analysis

The interrater reliability (IRR) and consistency of the panellist responses were analysed to ensure consensus. For calculation of the agreement percentage, the 5-Likert scale responses were dichotomized, with a score of 5 (strongly agree) and 4 (agree) representing agreement, and scores of 3 (neither agree or disagree), 2 (disagree) and 1 (strongly disagree) representing disagreement. The IRR and consistency of the criteria within the expert panel were evaluated using Cronbach’s α^37^. Continuous and dichotomous variables were reported as median (i.q.r.), mean(s.d.), and absolute and relative frequencies as appropriate.

## Results

**Fig. 1:**
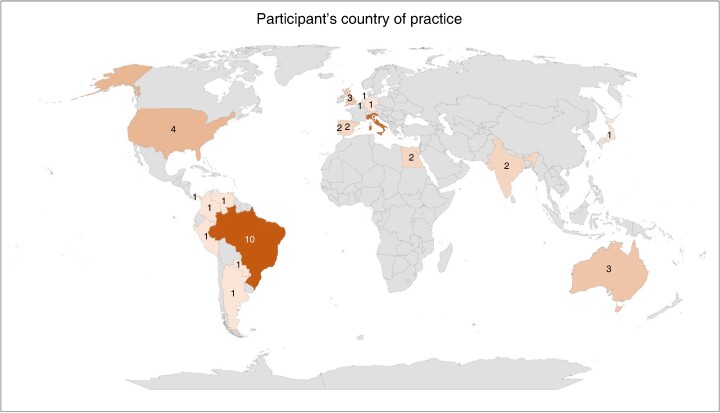
Demographic distribution of panellists

In the first round of the consensus approach, the survey was e-mailed to 218 experts and 47 responses were received (21.5%). Median responder age was 48 (i.q.r. 41–53) years, with 3 (6.4%), 13 (27.7%), 5 (10.6%), 12 (25.5%) and 13 (29.8%) of those surveyed endorsing 5–9, 10–14, 15–19, 20–24 and ≥25 years in clinical practice respectively. In terms of specialty, 26 (55.3%), 11 (23.4%), 6 (12.8%) and 4 (8.5%) were from urology, surgical oncology, general surgery and gynaecologic oncology respectively. In terms of setting, 32 (68%) were academic, followed by 7 (14.8%) community-based, 3 (6.4%) community-based university-affiliated and 5 (10.8%) private practice. The most common countries of practice among the participants were Italy (19.6%) and Brazil (19.6%). For more details regarding the participant’s country of practice, see *Fig. 1*.

Based on their expertise, 31 (66%), 10 (21.3%) and 22 (46.8%) of the participants treat penile cancer, vulval cancer and melanoma using ILND respectively. Median annual ILND surgical volume performed by surveyed experts was 10 (i.q.r. 5–20) cases. Various experts used multiple ILND surgical approaches as part of the treatment for penile cancer, vulval cancer or melanoma, including an open approach (91.5%), followed by laparoscopic/video endoscopic inguinal lymph node dissection (VEIL) (36.2%) and robotic (R-VEIL) (23.4%).

Of the experts, 91.4% (43 of 47) either agreed or strongly agreed with the importance of classifying and standardizing ILND perioperative complications into intraoperative and postoperative, and subclassifying postoperative into immediate (0–24 h), early (1–30 days) and late (31–90 days), and define them according to existing classification systems^19–21^. The experts were then surveyed on how to group complications. Of the presented list of intraoperative complications during ILND, 89.4% (42 of 47) agreed with the provided definitions and inclusion as part of the ILND intraoperative complication group; 93.6% (44 of 47) agreed that postoperative complications should be subclassified into five macrocategories. The survey showed agreement for the five macrocategories: 87.2% (41 of 47), 89.4% (42 of 47), 93.6% (44 of 47), 93.6% (44 of 47) and 100% (47 of 47) agreement for infectious, cutaneous, lymphatics, vascular and functional respectively. Experts were surveyed regarding their level of agreement with each complication definition and inclusion with the appropriate postoperative macrocategory. For more details regarding each complication definition and inclusion, see *Supplementary material appendices*.

Despite reaching a consensus on all items surveyed, several amendments were made after reviewing suggestions and feedback. *Supplementary material Table S1* highlights the main amendments to the Delphi survey following the first-round feedback.

**Fig. 2:**
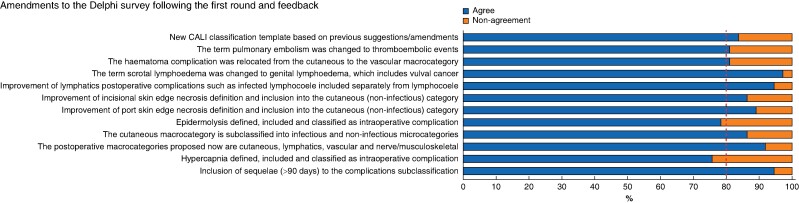
Sample round 2 results following the first round and feedback CALI, complications and adverse events in lymphadenectomy of the inguinal area.

The second round of the survey involved rating the agreement of the amendments made based on comments from the first round (*Supplementary material Table S1*). Thirty-seven of the 47 initial experts (78.7%) responded in the second round. All the amendments surpassed the minimum of greater than 80% agreement (agree or strongly agree), except for two items: whether ‘hypercapnia should be classified as an intraoperative complication, defined and included’ and whether ‘epidermolysis should be classified, defined and included within cutaneous macrocategory,’ which reached 75.7% (28 of 37) and 78.4% (29 of 37) agreement respectively. Of note, a consensus was reached for these two items in the first round. However, based on comments, they were assessed again in the second round, and here did not pass the 80% threshold (*Fig. 2*).

The main improvements agreed upon during the second round were the inclusion of sequelae (>90 days) to the complication subclassification system (95% agreement, 35 of 37) and restructuring macrocategories based on location/system, such as cutaneous (86.5% agreement, 32 of 37), lymphatics (95% agreement, 35 of 37), vascular (81% agreement, 30 of 37) and nerve/musculoskeletal (95% agreement, 35 of 37), rather than mixed aetiology and site of complication that was proposed before. The inclusion of infectious and non-infectious microcategories was included within the cutaneous macrocategory. *Supplementary material Table S2* highlights the main amendments to the Delphi survey following the second round and feedback. For more details regarding each complication definition and inclusion, see *Supplementary material appendices*. Cronbach’s α for the second round of the Delphi process was 0.88 (indicating good IRR agreement^37^).

**Fig. 3:**
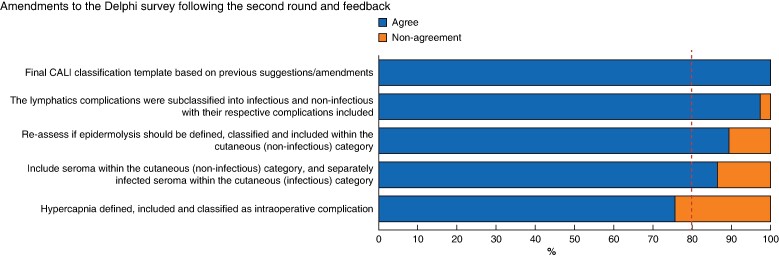
Sample round 3 results following the second round and feedback CALI, complications and adverse events in lymphadenectomy of the inguinal area.

The third and final round consisted of assessing the rate of agreement for the two items not surpassing the threshold in round two and amendments made based on feedback from the second round (*Supplementary material Table S2*). All 37 experts (100%) that participated in previous rounds responded in this round. Consensus was reached on all surveyed items. This round’s main amendment was the inclusion of infectious and non-infectious microcategories with their respective complications within the lymphatics macrocategory (97.3% agreement, 36 of 37). These microcategories were re-affirmed within the cutaneous macrocategory (86.5% agreement, 32 of 37) (*Fig. 3*).

**Fig. 4:**
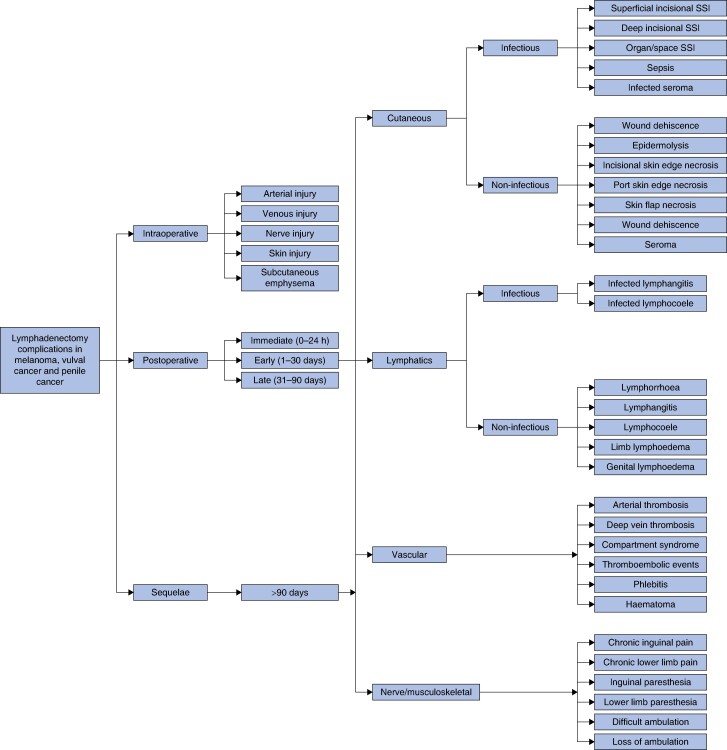
Standardized classification system for reporting ILND complications in melanoma, vulval cancer and penile cancer ILND, inguinal lymph node dissection; SSI, surgical site infection.

Unanimous agreement (100%, 37 of 37) was achieved with the final standardized classification system for reporting ILND complications in melanoma, vulval cancer and penile cancer (*Fig. 4*). IRR agreement was higher compared with the previous round (Cronbach’s α: 0.90).

## Discussion

In this study, a new CALI classification system to report complications associated with ILND was created. The CALI group’s end goal is to help decrease the morbidity rate associated with this procedure and to provide a standardized system to classify and report perioperative complications using common and widely accepted terminology.

A previous systematic review demonstrated that classification systems are not properly utilized in the literature^1^. Despite the existence of several published guidelines to standardize complication reporting, only 25% of studies report at least half of the minimum requirements for complication reporting^1,17,27,38^, highlighting a need for improvement.

Improving outcomes associated with ILND has several challenges. First, it is performed in the setting of rare diseases; hence, collecting a large number of patients to boost the power of the studies is difficult. Secondly, the centres of expertise are sparse, so the definitions of complications are highly variable in the literature and are mostly based on the surgeon’s personal experiences. Therefore, having a standardized system for classifying ILND complications and AEs will enable an increased body of data on this topic that can inform targets to improve patient outcomes.

This classification has several macrocategories for postoperative AEs based on location/system (cutaneous, lymphatics, vascular and nerve/musculoskeletal). Additionally, the cutaneous and lymphatics macrocategories were subclassified into infectious and non-infectious microcategories to avoid overlapping classifications. The categories were initially proposed and further refined through surveying experts. Theoretically, the macrocategories will aid in identifying common aetiologies for several complications. For instance, evidence suggests that a minimally invasive approach decreases the rate of infectious and cutaneous complications, but it is less clear if the lymphatic system morbidity rate was impacted^1,11,16,39–41^. The development of targeted interventions for identifying lymphatic leaks and assessing the impact of advanced energy devices is needed^42–45^.

An advantage of this system is that microcategory definitions were clear and standardized. For example, terms like ‘wound dehiscence’ and ‘skin flap necrosis’^46^ can be used interchangeably in the literature, leading to misinterpretation of true incidence.

There are limitations to this consensus study. Although this classification system was developed through surveying experts from different specialties that use different surgical approaches, it might not be representative of all surgeons. Initially, the assessment will focus on quantifying IRR across various expertise strata, followed by an external validation phase to confirm the system’s applicability and reliability in diverse clinical environments. This phase represents a pivotal progression towards the prospective application of the classification in studies related to ILNDs, with potential substantial implications for the field.

## Supplementary Material

zrae056_Supplementary_Data

## Data Availability

The authors confirm that the data supporting the findings of this study are available within the article.
