# Erratum to: Complications and adverse events in lymphadenectomy of the inguinal area: worldwide expert consensus

**DOI:** 10.1093/bjsopen/zrae112

**Published:** 2024-08-14

This is an erratum to: René Sotelo *et al.*, Complications and adverse events in lymphadenectomy of the inguinal area: worldwide expert consensus, *BJS Open*, Volume 8, Issue 4, August 2024, zrae056, https://doi.org/10.1093/bjsopen/zrae056

This article has been corrected to indicate that the author Humberto M. Pontillo is affiliated with the Department of Surgery at the Sant Jaume of Calella Hospital, Barcelona, Spain, and not with the Department of Urology at the same institution, as originally stated.

