# Supplementary material for: Complications and adverse events in lymphadenectomy of the inguinal area: worldwide expert consensus
Source: BJS Open. 2024 Jul 11;8(4):zrae056. doi: 10.1093/bjsopen/zrae056 (PMC11236483; doi:10.1093/bjsopen/zrae056)
Supplement: zrae056_Supplementary_Data [file zrae056_supplementary_data.zip › Supplementary material_Table 1.docx]

| Amendments to the Delphi survey following the first round and feedback | |
| --- | --- |
| # | **Amendments** |
| 1 | Inclusion of sequelae (>90 days) to the complications sub-classification. |
| 2 | Postoperative macro-categories were re-structured based on location/system rather than mixed etiology and site of complication that was proposed before.  The postoperative macro-categories proposed now are Cutaneous, Lymphatics, Vascular, and Nerve/Musculoskeletal. |
| 3 | The “infectious” complications were included and sub-classified within the cutaneous macro-category.  The cutaneous macro-category is sub-classified into infectious and non-infectious micro-categories |
| 4 | Improvement of “port skin edge necrosis” definition and inclusion into the cutaneous (non-infectious) category. |
| 5 | Improvement of “incisional skin edge necrosis” definition and inclusion into the cutaneous (non-infectious) category. |
| 6 | Improvement of lymphatics postoperative complications such as infected lymphocele included separately from lymphocele. |
| 7 | The term scrotal lymphedema was changed into genital lymphedema, which encloses vulvar cancer. |
| 8 | The hematoma complication was relocated from the cutaneous to the vascular macro-category. |
| 9 | The term pulmonary embolism was changed into thromboembolic events. |
| 10 | New CALI classification template based on previous suggestions/amendments. |

***Table 1.*** Amendments to the Delphi survey following the first round and panelists' feedback.
