# Supplementary material for: Complications and adverse events in lymphadenectomy of the inguinal area: worldwide expert consensus
Source: BJS Open. 2024 Jul 11;8(4):zrae056. doi: 10.1093/bjsopen/zrae056 (PMC11236483; doi:10.1093/bjsopen/zrae056)
Supplement: zrae056_Supplementary_Data [file zrae056_supplementary_data.zip › Supplementary material_Table 2.docx]

***Table 2.*** Amendments to the Delphi survey following the second round and panelists' feedback.

| Amendments to the Delphi survey following the second round and feedback | |
| --- | --- |
| # | **Amendments** |
| 1 | Consider “hypercapnia” as an anesthesia-related complication and not related to ILND. Therefore, exclude from the classification. |
| 2 | Include “seroma” within the cutaneous (non-infectious) category, and separately “infected seroma” within the cutaneous (infectious) category. |
| 3 | Re-assess if “epidermolysis” should be defined, classified, and included within the cutaneous (non-infectious) category. |
| 4 | The “lymphatics” complications were sub-classified into infectious and non-infectious with their respective complications included |
| 5 | Final CALI classification template based on previous suggestions/amendments. |
