# Supplementary material for: Complications and adverse events in lymphadenectomy of the inguinal area: worldwide expert consensus
Source: BJS Open. 2024 Jul 11;8(4):zrae056. doi: 10.1093/bjsopen/zrae056 (PMC11236483; doi:10.1093/bjsopen/zrae056)
Supplement: zrae056_Supplementary_Data [file zrae056_supplementary_data.zip › Supplementary-Material-Template.docx]

**Complications and adverse events in lymphadenectomy of the inguinal area (CALI) - A worldwide expert consensus**

René Sotelo^1,*^, Aref S. Sayegh^1,2^, Luis G. Medina^1^, Laura C. Perez^1,3^, Anibal La Riva^1,4^, Michael B. Eppler^1^, José Gaona^5^, Marcos Tobias-Machado^6^, Philippe E. Spiess^7^, Curtis A. Pettaway^8^, Antonio Carlos Lima Pompeo^9^, Pablo Aloisio Lima Mattos^10^, Timothy G. Wilson^11^, Gustavo M. Villoldo^12^, Eric Chung^13^, Aldo Samaniego^14^, Antonio Augusto Ornellas^15^, Vladimir Pinheiro^16^, Eder S. Brazão Jr.^16^, David Subira-Rios^17^, Leandro Koifman^18^, Stênio de Cassio Zequi ^19^, Humberto M. Pontillo Z^20^, José de Ribamar Rodrigues Calixto^21^, Rafael Campos Silva^22^, B. Mark Smithers^23^, Simone Garzon^24^, Oliver Haase^25^, Antonio Sommariva^26^, Robert Fruscio^27^, Francisco Martins^28^, Pedro S. de Oliveira^28^, Giovanni Battista Levi Sandri^29^, Marco Clementi^30^, Juan Astigueta^31^, Islam H. Metwally^32^, Rasiah Bharathan^33^, Tarun Jindal^34^, Yasuhiro Nakamura^35^, Hisham Abdel Mageed^36^, Sakthiushadevi Jeevarajan^37^, Ramón Rodriguez Lay^38^, Herney Andrés García-Perdomo^39^, Omaira Rodríguez González^40^, Saum Ghodoussipour^41^, Inderbir Gill^1^, Giovanni E. Cacciamani^1^

1. Catherine and Joseph Aresty Department of Urology, USC Institute of Urology, Keck School of Medicine, University of Southern California, Los Angeles, CA, USA.
2. Department of Surgery, MedStar Franklin Square Medical Center, Baltimore, Maryland, USA.
3. Department of Surgery, Johns Hopkins University School of Medicine, Baltimore, Maryland, USA.
4. Department of General Surgery, Digestive Disease & Surgery Institute, Cleveland Clinic Foundation, Cleveland, Ohio, USA.
5. Universidad de Santander, Instituto Uromédica, Bucaramanga, Colombia.
6. Department of Urology, Instituto do Câncer Arnaldo Vieira de Carvalho, São Paulo, Brazil.
7. Department of GU Oncology and Tumor Biology, Moffitt Cancer Center, Tampa, FL, USA.
8. The University of Texas, M.D. Anderson Cancer Center, Houston, TX, USA.
9. Faculdade de Medicina do ABC, São Paulo, Brazil.
10. Associação Piauiense de Combate ao Câncer, Teresina, PI, Brazil.
11. Providence St. John’s Cancer Institute, Santa Monica, CA, USA.
12. Department of Urology, Instituto Alexander Fleming, Buenos Aires, Argentina.
13. Department of Urology, Princess Alexandra Hospital, University of Queensland, Brisbane QLD Australia.
14. Servicio de Urología del Hospital Central del Instituto de Previsión Social, Asunción, Paraguay.
15. Departamento de Urologia, Instituto Nacional do Câncer do Brasil (INCA), RJ, Brazil.
16. Department of Urology, AC Camargo Cancer Center, São Paulo, Brazil.
17. Urology Department, Gregorio Marañon Universitary Hospital. Madrid. Spain.
18. Serviço de Urologia, Hospital Municipal Souza Aguiar, Rio de Janeiro, RJ, Brazil.
19. Department of Urology-AC Camargo Cancer Center-São Paulo-São Paulo, Brazil/ National Institute for Science and Technology in Oncogenomics and Therapeutic Innovation, São Paulo, Brazil/ Graduate School of Urology-Escola Paulista de Medicina-Universidade Federal de São Paulo, Brazil.
20. Sant Jaume of Calella Hospital, Barcelona, Spain.
21. Department of Medicine II, Federal University of Maranhão, São Luís, MA, Brazil.
22. Hospital Universitário Presidente Dutra - HUPD/UFMA, Brazil.
23. University of Queensland, Queensland Melanoma Project, Princess Alexandra Hospital, Brisbane, Australia.
24. Department of Surgery, Dentistry, Pediatrics, and Gynecology, University of Verona, Verona, Italy.
25. University Medicine Berlin - Charité, Department of Surgery, Germany.
26. Veneto Institute of Oncology IOV-IRCCS Padova Italy.
27. Department of Medicine and Surgery, University of Milan Bicocca, ASST Monza, Italy.
28. Department of Urology, Centro Hospitalar Universitário Lisboa Norte, Hospital de Santa Maria, Lisbon, Portugal.
29. Department of Surgery, ASL Frosinone, Italy.
30. Department of Medicine, Health and Life, University of L'Aquila, Piazza S. Tommasi, 67100 L'Aquila, AQ, Italy.
31. Universidad Privada Antenor Orrego. Trujillo. Perú.
32. Surgical Oncology Department, Oncology Center Mansoura University (OCMU), Mansoura, Egypt.
33. Department of Gynaecological Oncology, Medical University of Vienna, Austria.
34. Department of Uro-oncology, Narayana Super Speciality Hospital, Howrah, India.
35. Department of Skin Oncology/Dermatology, Saitama Medical University International Medical Center, Saitama, Japan.
36. National Cancer Institute Cairo University, Cairo, Egypt.
37. Department of Surgical Oncology, Regional Cancer Centre, Kanchipuram, Tamil Nadu, India.
38. Clinic Urology Service, Complejo Hospitalario Metropolitano, Caja de Seguro Social Dr. Arnulfo Arias Madrid, Panama.
39. Division of Urology/Urooncology. Department of Surgery. School of Medicine. Universidad del Valle. Colombia.
40. Chief of Surgical Department. Clínicas Caracas Hospital. Faculty of Medicine. Central University of Venezuela. Caracas, Venezuela.
41. Sections of Urologic Oncology, Rutgers Cancer Institute of New Jersey and Rutgers Robert Wood Johnson Medical School, New Brunswick, NJ, USA

**Corresponding author**

Rene Sotelo, M.D.

USC Institute of Urology, Keck School of Medicine

University of Southern California, Los Angeles, CA, USA

Email: rene.sotelo@med.usc.edu

Phone: 323-865-3700

ORCID: 0000-0002-8580-8476

Twitter: @doctorsotelo

**Supplementary Materials - Index**

| **Supplementary Methods** |  |
| --- | --- |
| ACCORD checklist | *page 3-5* |
| **Supplementary Appendixes** |  |
| Detailed complications definitions and inclusion category | *page 6-24* |
| **Supplementary Figures and Tables** |  |
| Table 1 | *page 25* |
| Table 2 | *page 26* |
|  |  |

**Supplementary Methods**


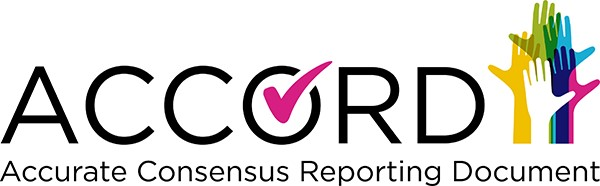


| Item No. | Section | Checklist Item (*help text*) | Page No. |
| --- | --- | --- | --- |
| T1 | **Title** | Identify the article as reporting a consensus exercise and state the consensus methods used in the title.  *For example, Delphi or nominal group technique.* | 1 |
| I1 | **Introduction** | Explain why a consensus exercise was chosen over other approaches. | 5 |
| I2 |  | State the aim of the consensus exercise, including its intended audience and geographical scope (national, regional, global). | 5 |
| I3 |  | If the consensus exercise is an update of an existing document, state why an update is needed, and provide the citation for the original document. | - |
| M1 | **Methods**  Registration | If the study or study protocol was prospectively registered, state the registration platform and provide a link. If the exercise was not registered, this should be stated.  *Recommended to include the date of registration.* | 5 |
| M2 | Selection of SC and/or panellists | Describe the role(s) and areas of expertise or experience of those directing the consensus exercise.  *For example, whether the project was led by a chair, co-chairs or a steering committee, and, if so, how they were chosen. List their names if appropriate, and whether there were any subgroups for individual steps in the process.* | 5-6 |
| M3 |  | Explain the criteria for panellist inclusion and the rationale for panellist numbers. State who was responsible for panellist selection. | 5-6 |
| M4 |  | Describe the recruitment process (how panellists were invited to participate).  *Include communication/advertisement method(s) and locations, numbers of invitations sent, and whether there was centralised oversight of invitations or if panellists were asked/allowed to suggest other members of the panel.* | 6 |
| M5 |  | Describe the role of any members of the public, patients or carers in the different steps of the study. | 6-7 |
| M6 | Preparatory research | Describe how information was obtained prior to generating items or other materials used during the consensus exercise.  *This might include a literature review, interviews, surveys, or another process.* | 5-6 |
| M7 |  | Describe any systematic literature search in detail, including the search strategy and dates of search or the citation if published already.  *Provide the details suggested by the reporting guideline PRISMA and the related PRISMA-Search extension.* | 5-6 |
| M8 |  | Describe how any existing scientific evidence was summarised and if this evidence was provided to the panellists. | 5-6 |
| M9 | Assessing consensus | Describe the methods used and steps taken to gather panellist input and reach consensus (for example, Delphi, RAND-UCLA, nominal group technique).  *If modifications were made to the method in its original form, provide a detailed explanation of how the method was adjusted and why this was necessary for the purpose of your consensus-based study.* | 5-6 |
| M10 |  | Describe how each question or statement was presented and the response options. State whether panellists were able to or required to explain their responses, and whether they could propose new items.  *Where possible, present the questionnaire or list of statements as supplementary material.* | 6 |
| M11 |  | State the objective of each consensus step.  *A step could be a consensus meeting, a discussion or interview session, or a Delphi round.* | 6-7 |
| M12 |  | State the definition of consensus (for example, number, percentage, or categorical rating, such as ‘agree’ or ‘strongly agree’) and explain the rationale for that definition. | 6-7 |
| M13 |  | State whether items that met the prespecified definition of consensus were included in any subsequent voting rounds. | 6 |
| M14 |  | For each step, describe how responses were collected, and whether responses were collected in a group setting or individually. | 6-7 |
| M15 |  | Describe how responses were processed and/or synthesised.  *Include qualitative analyses of free-text responses (for example, thematic, content or cluster analysis) and/or quantitative analytical methods, if used.* | 6-7 |
| M16 |  | Describe any piloting of the study materials and/or survey instruments.  *Include how many individuals piloted the study materials, the rationale for the selection of those individuals, any changes made as a result and whether their responses were used in the calculation of the final consensus. If no pilot was conducted, this should be stated.* | 6 |
| M17 |  | If applicable, describe how feedback was provided to panellists at the end of each consensus step or meeting.  *State whether feedback was quantitative (for example, approval rates per topic/item) and/or qualitative (for example, comments, or lists of approved items), and whether it was anonymised.* | 6 |
| M18 |  | State whether anonymity was planned in the study design. Explain where and to whom it was applied and what methods were used to guarantee anonymity. | - |
| M19 |  | State if the steering committee was involved in the decisions made by the consensus panel.  *For example, whether the steering committee or those managing consensus also had voting rights.* | 6 |
| M20 | Participation | Describe any incentives used to encourage responses or participation in the consensus process.  *For example, were invitations to participate reiterated, or were participants reimbursed for their time.* | 6 |
| M21 |  | Describe any adaptations to make the surveys/meetings more accessible.  *For example, the languages in which the surveys/meetings were conducted and whether translations or plain language summaries were available*. | - |
| R1 | Results | State when the consensus exercise was conducted. List the date of initiation and the time taken to complete each consensus step, analysis, and any extensions or delays in the analysis. | 6 |
| R2 |  | Explain any deviations from the study protocol, and why these were necessary.  *For example, addition of panel members during the exercise, number of consensus steps, stopping criteria; report the step(s) in which this occurred.* | 6-7 |
| R3 |  | For each step, report quantitative (number of panellists, response rate) and qualitative (relevant socio-demographics) data to describe the participating panellists. | 7-8 |
| R4 |  | Report the final outcome of the consensus process as qualitative (for example, aggregated themes from comments) and/or quantitative (for example, summary statistics, score means, medians and/or ranges) data. | 7-8 |
| R5 |  | List any items or topics that were modified or removed during the consensus process. Include why and when in the process they were modified or removed. | 7-8 |
| D1 | Discussion | Discuss the methodological strengths and limitations of the consensus exercise.  *Include factors that may have impacted the decisions (for example, response rates, representativeness of the panel, potential for feedback during consensus to bias responses, potential impact of any non-anonymised interactions).* | 9 |
| D2 |  | Discuss whether the recommendations are consistent with any pre-existing literature and, if not, propose reasons why this process may have arrived at alternative conclusions. | 8-9 |
| O1 | Other information | List any endorsing organisations involved and their role. | - |
| O2 |  | State any potential conflicts of interests, including among those directing the consensus study and panellists. Describe how conflicts of interest were managed. | 3, 10 |
| O3 |  | State any funding received and the role of the funder.  *Specify, for example, any funder involvement in the study concept/design, participation in the steering committee, conducting the consensus process, funding of any medical writing support. This could be disclosed in the methods or in the relevant transparency section of the manuscript. Where a funder did not play a role in the process or influence the decisions reached, this should be specified.* | 3, 10 |

**Supplementary Appendixes**

**
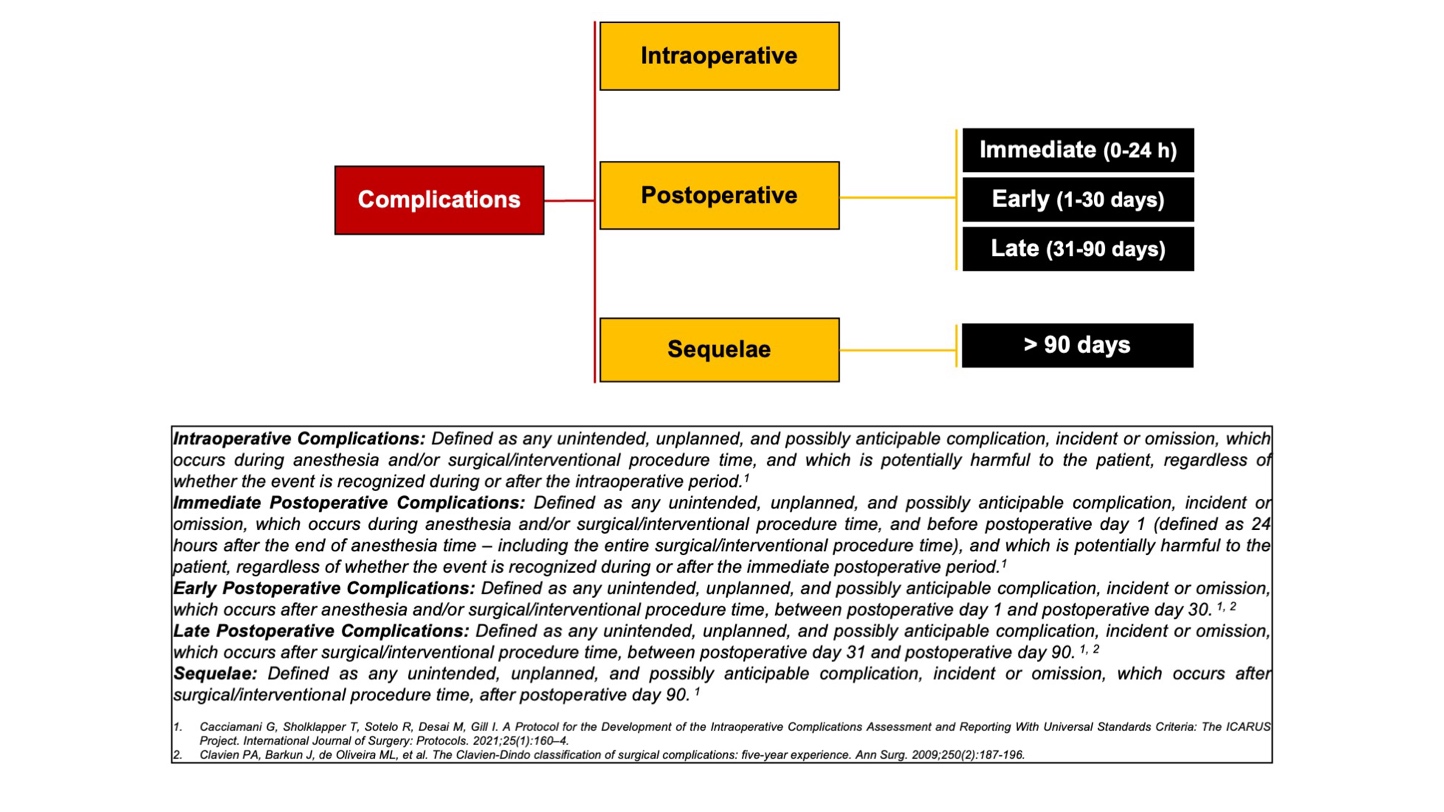
**

**
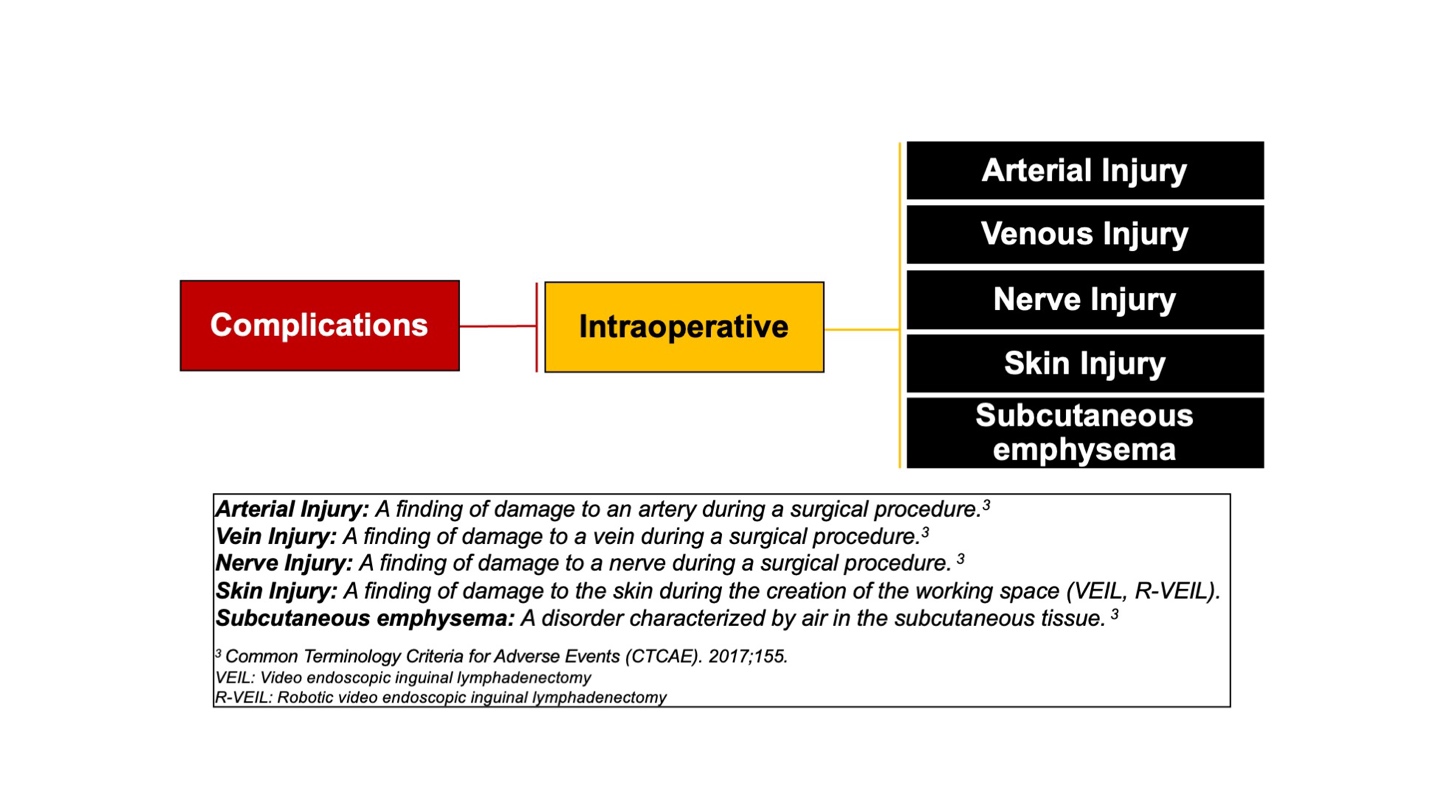
**

**
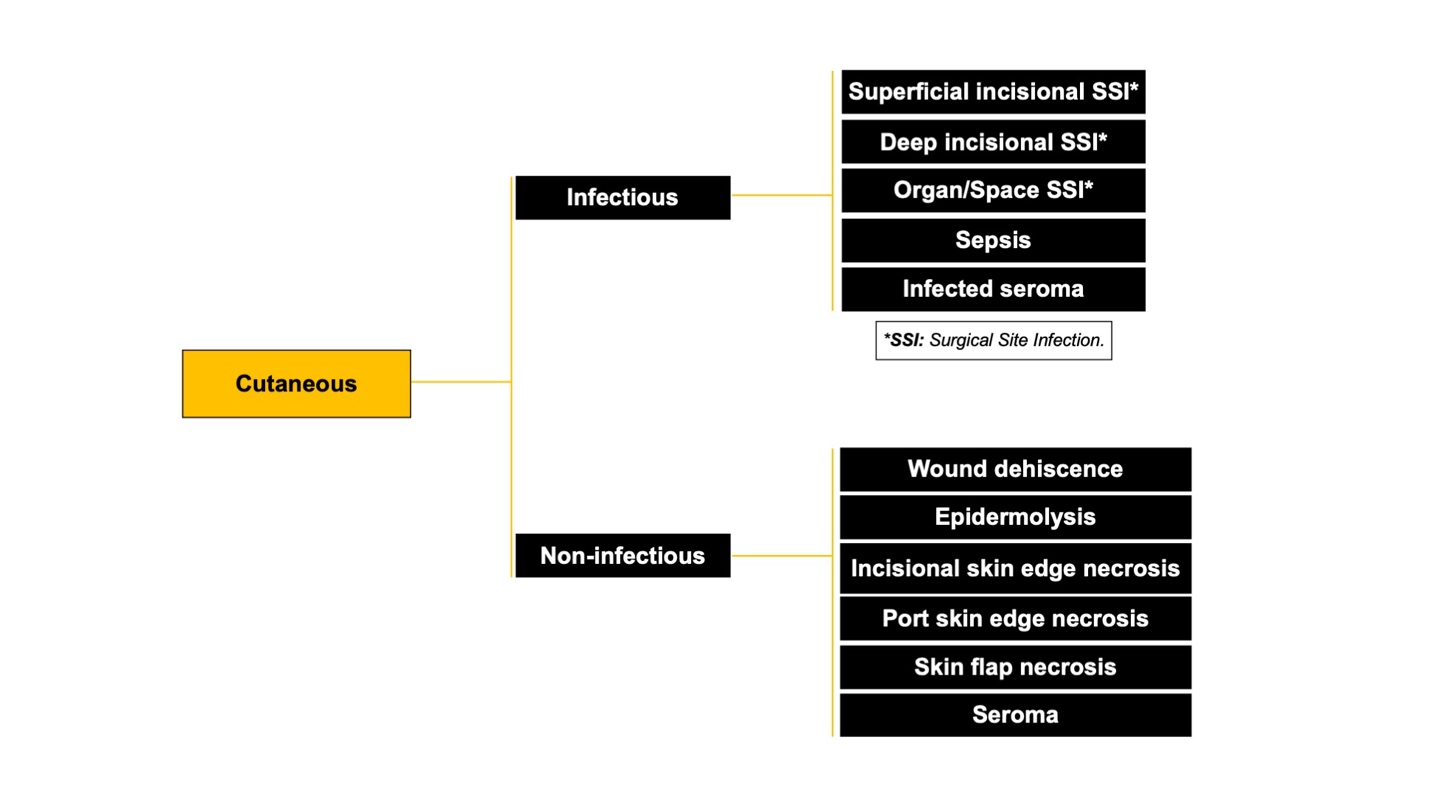

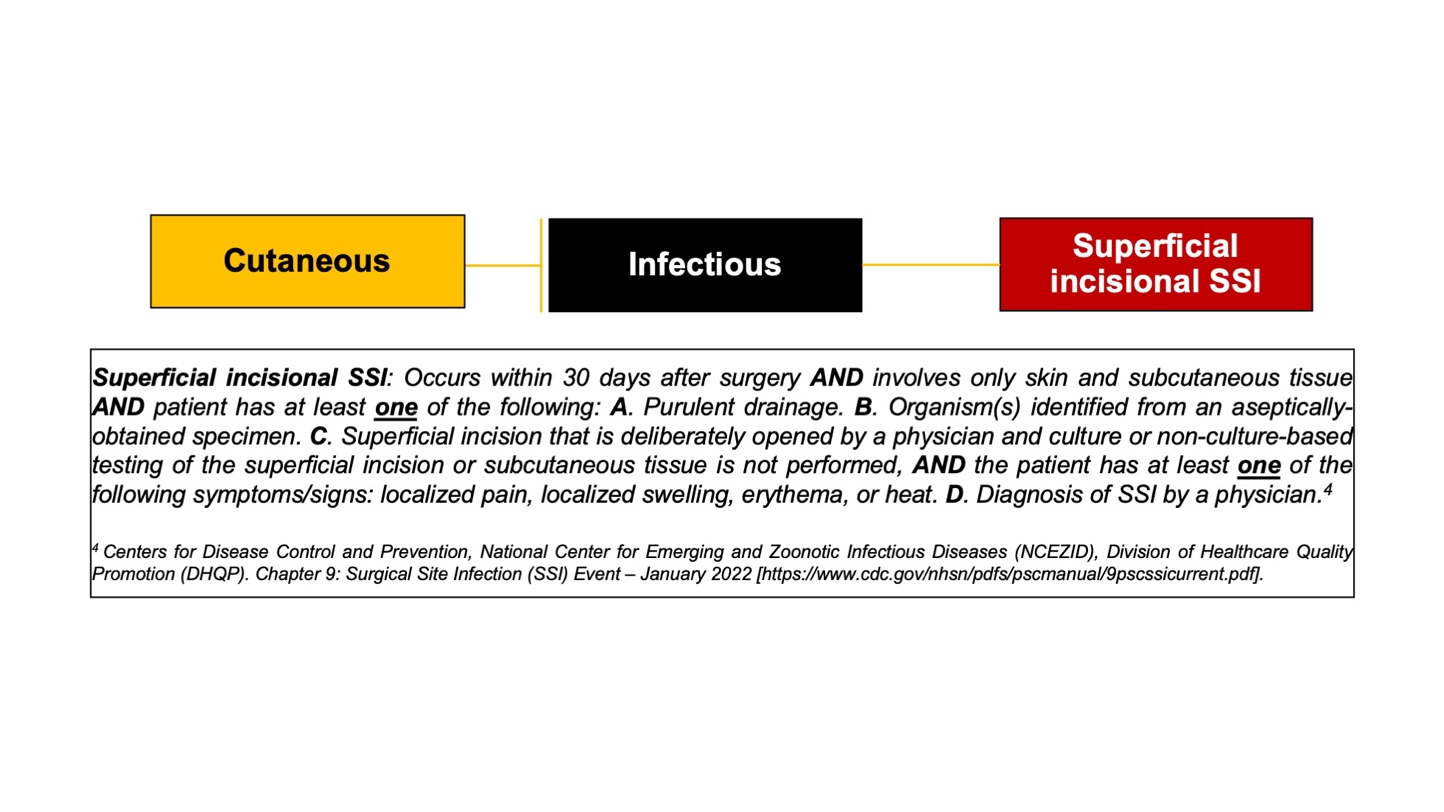

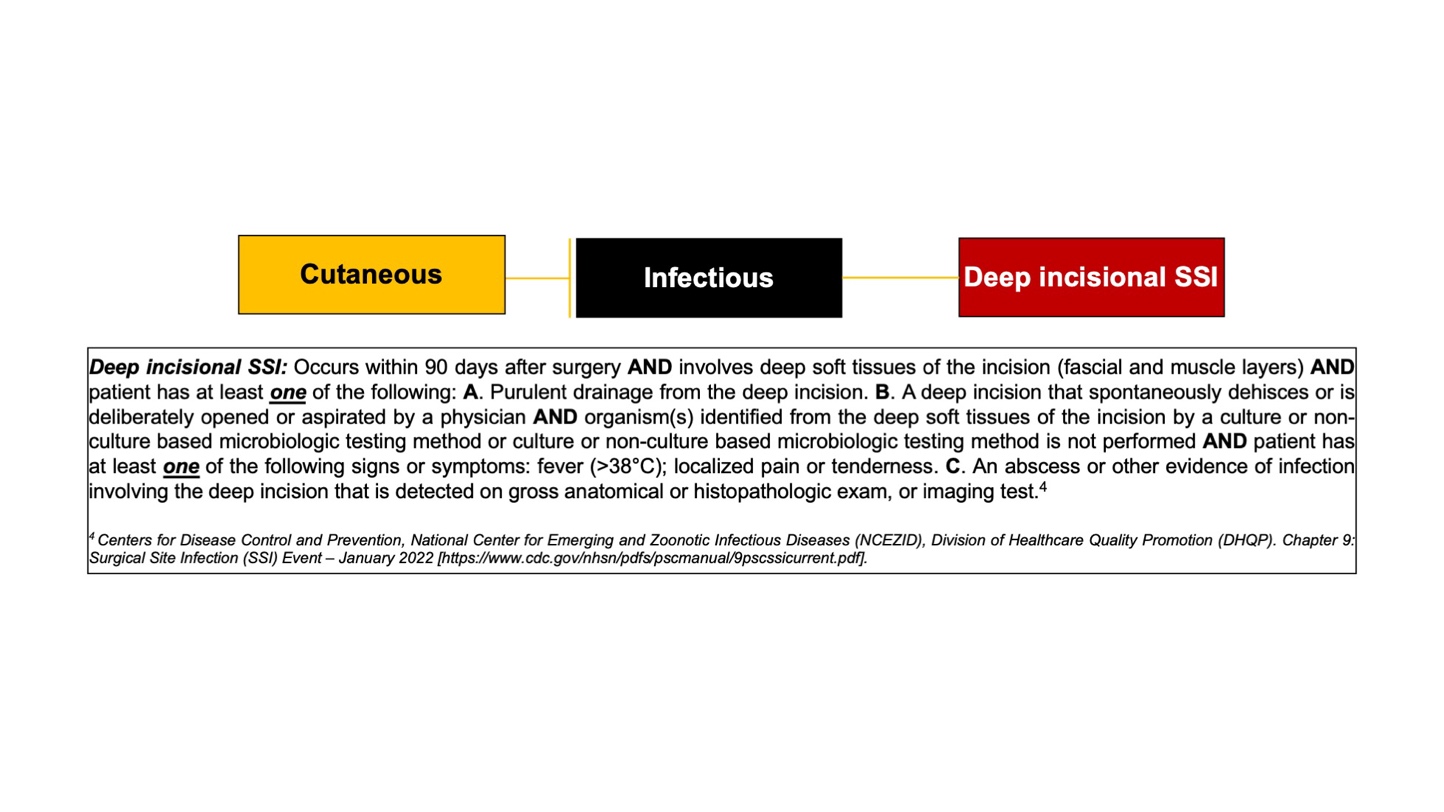

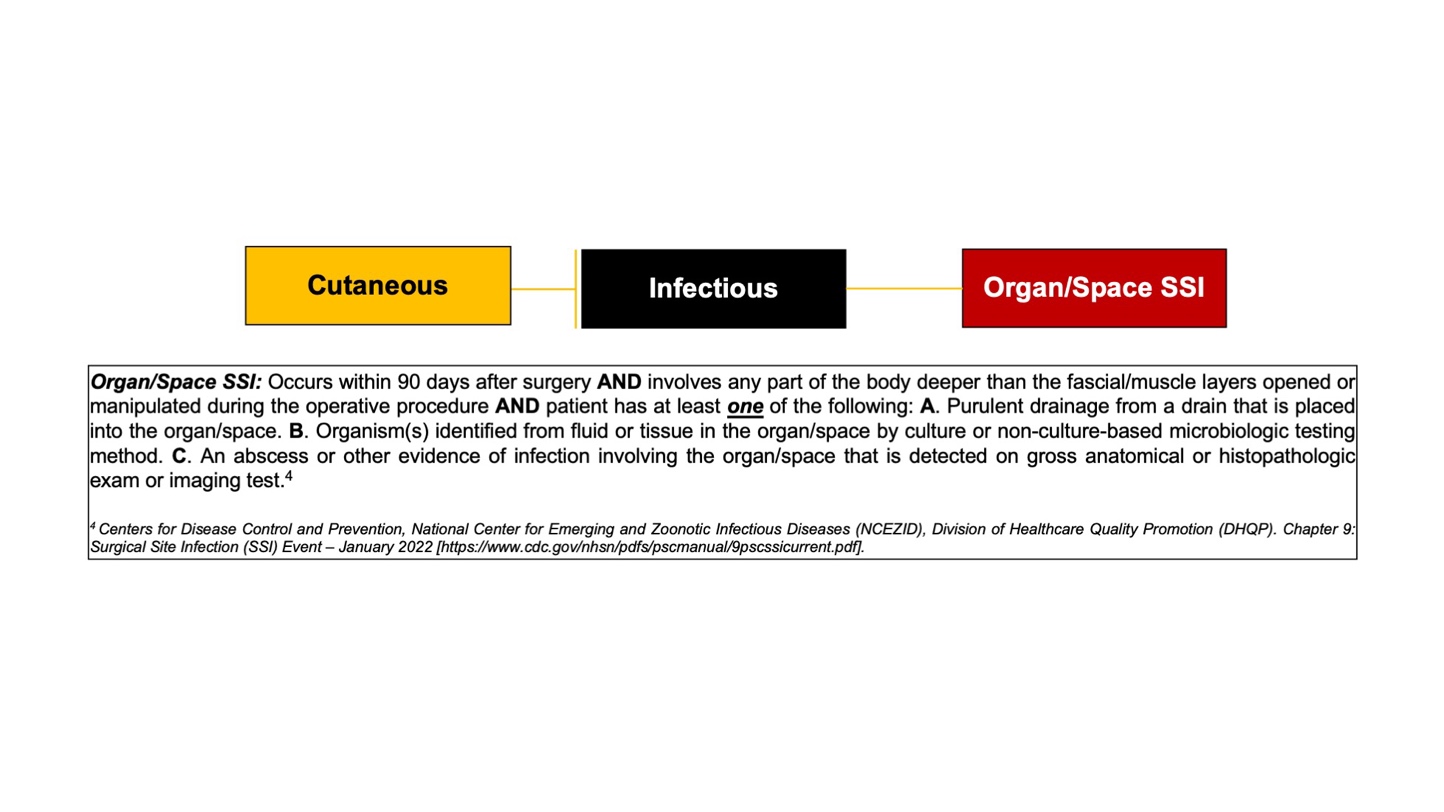

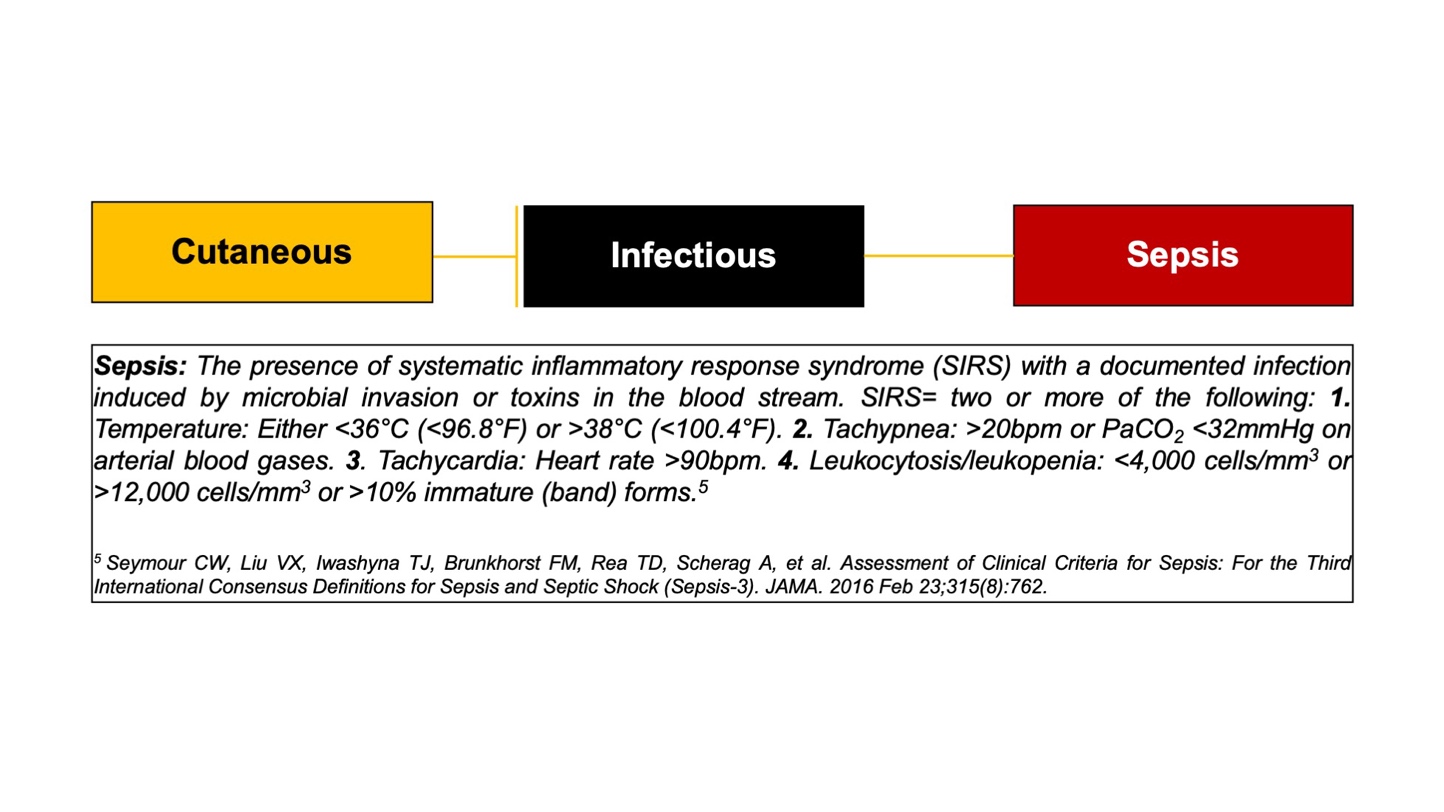

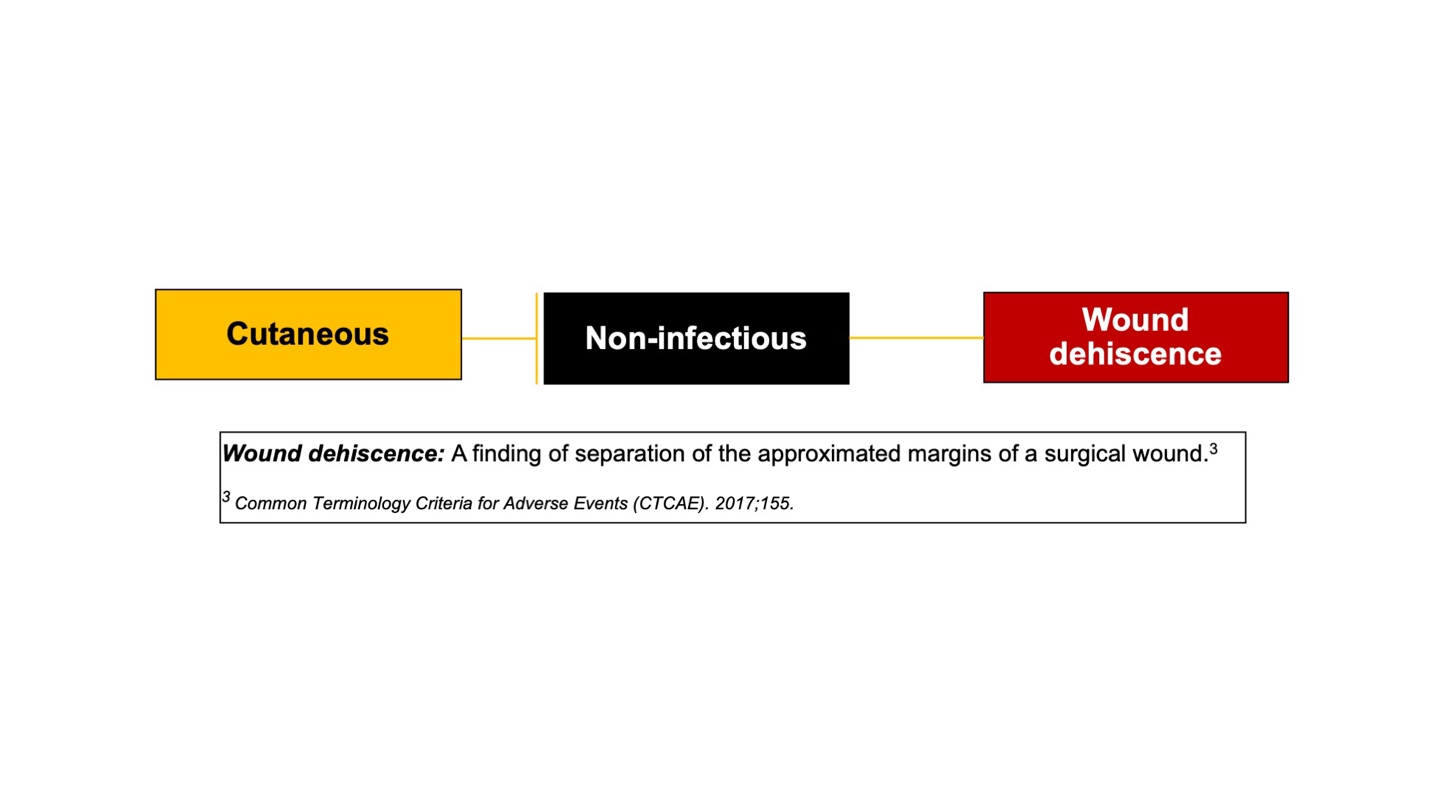

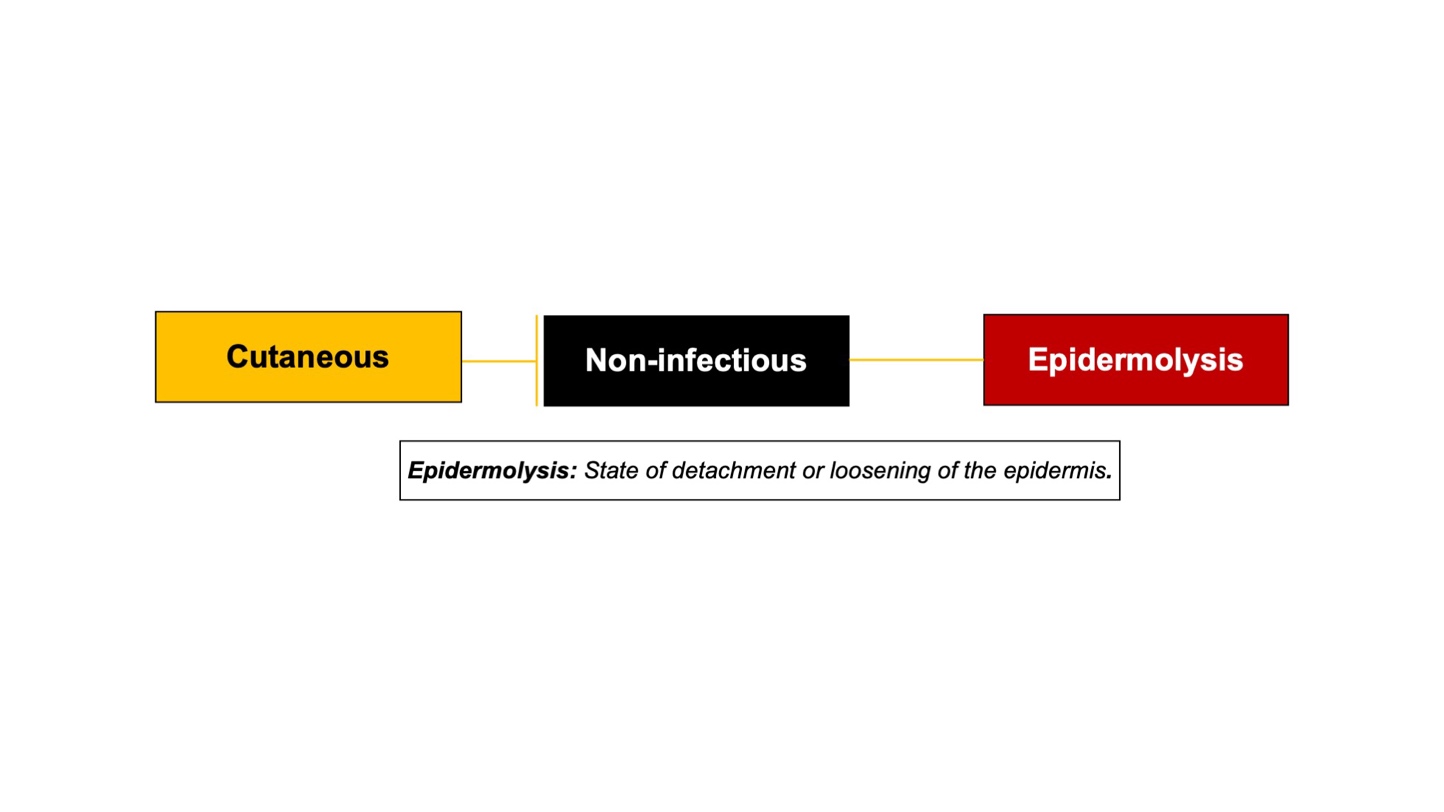

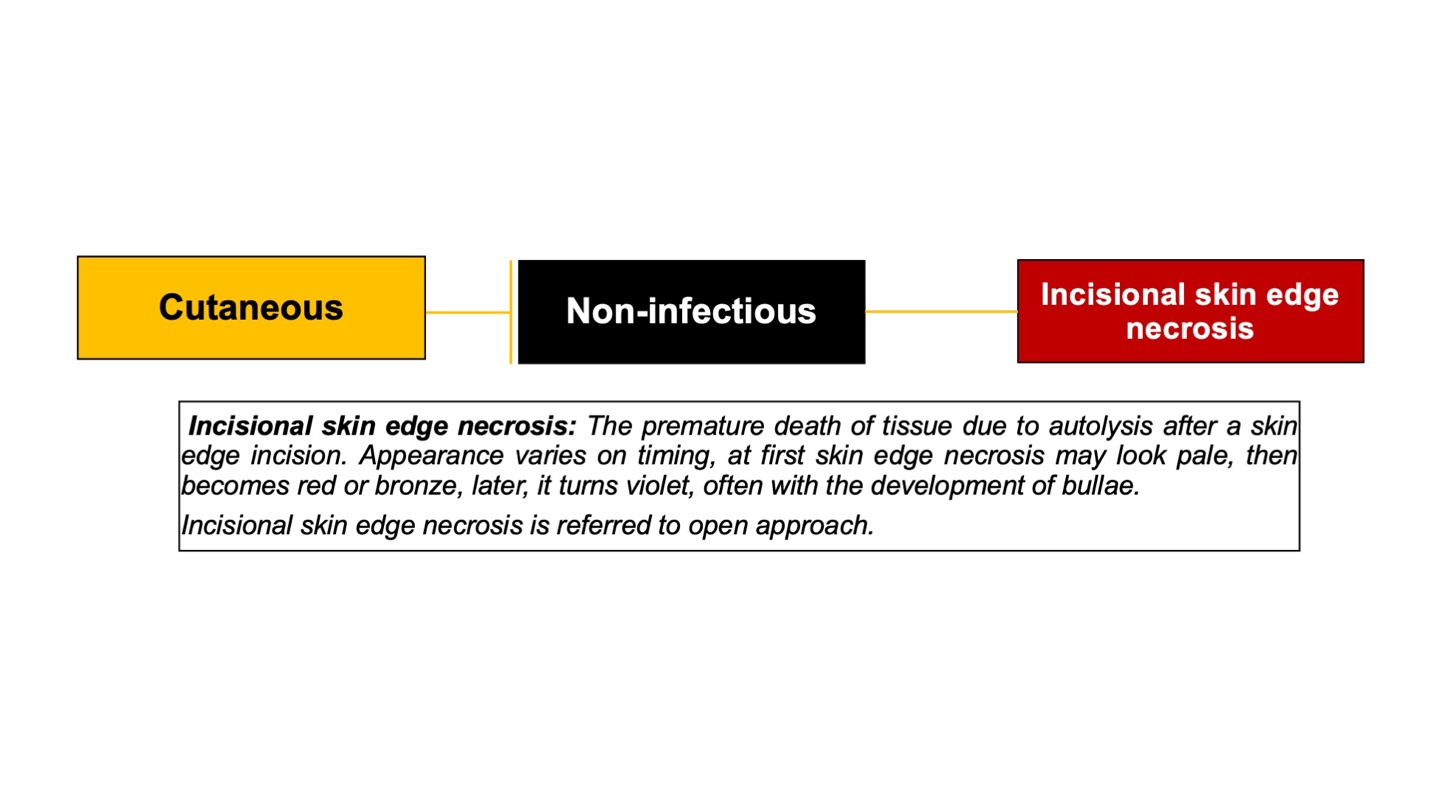

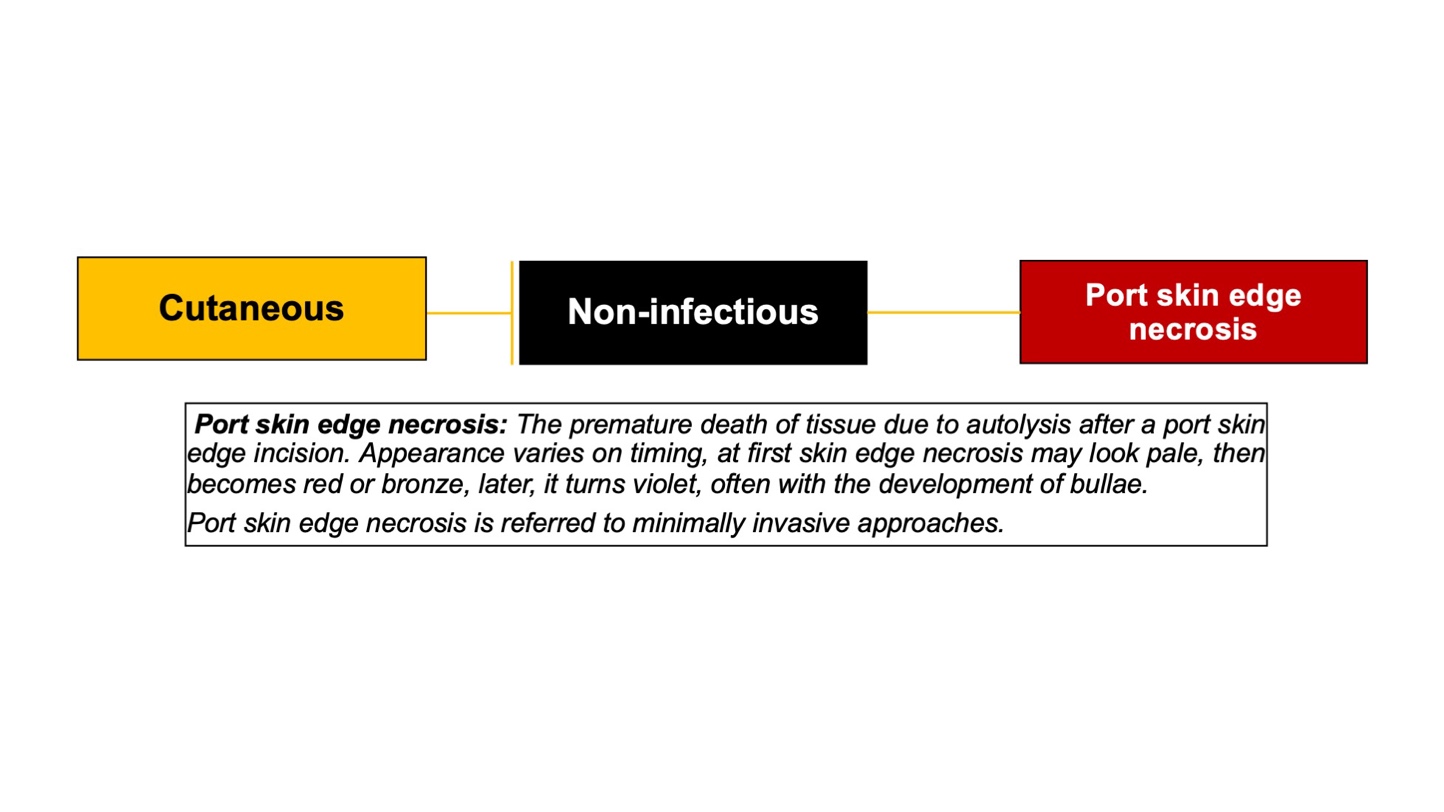

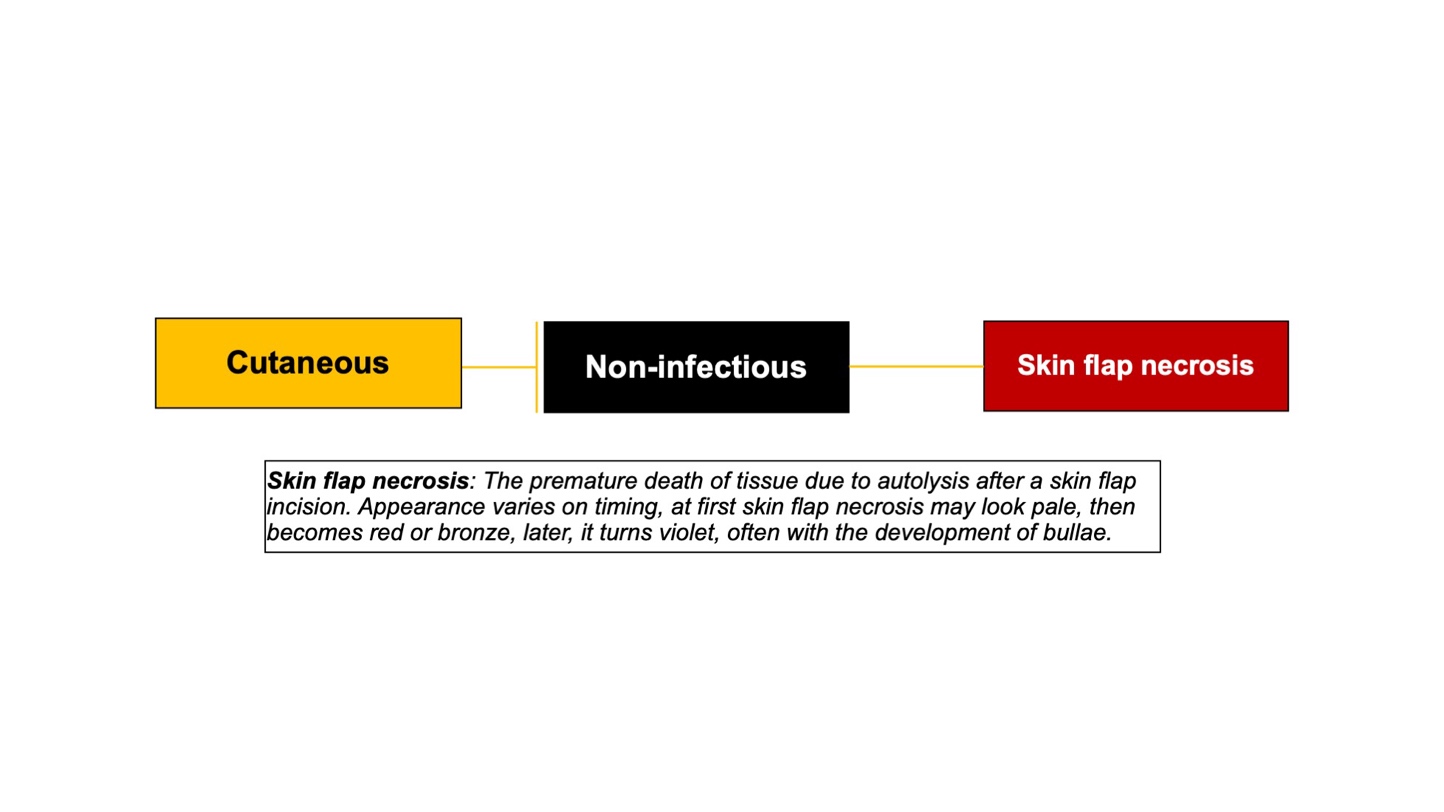

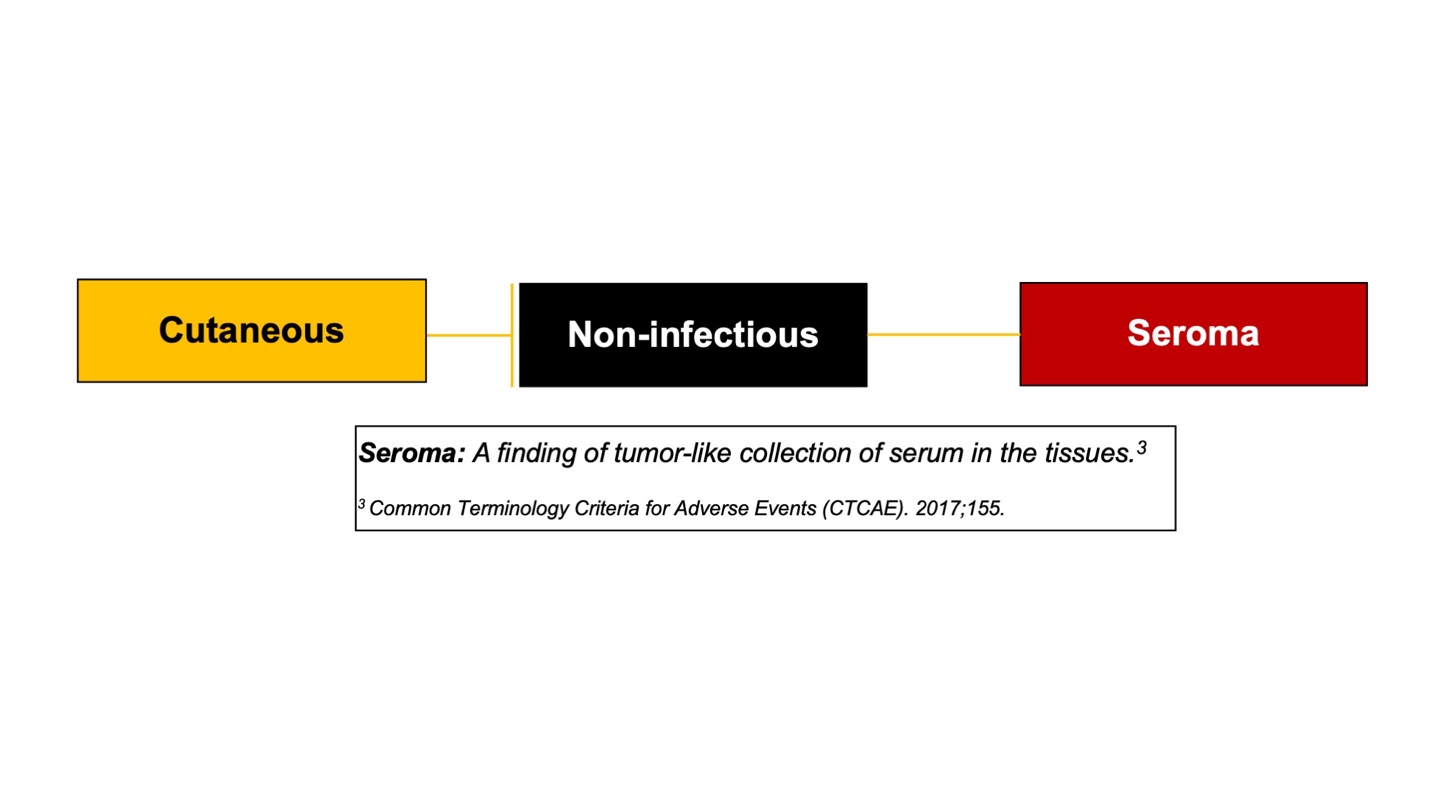

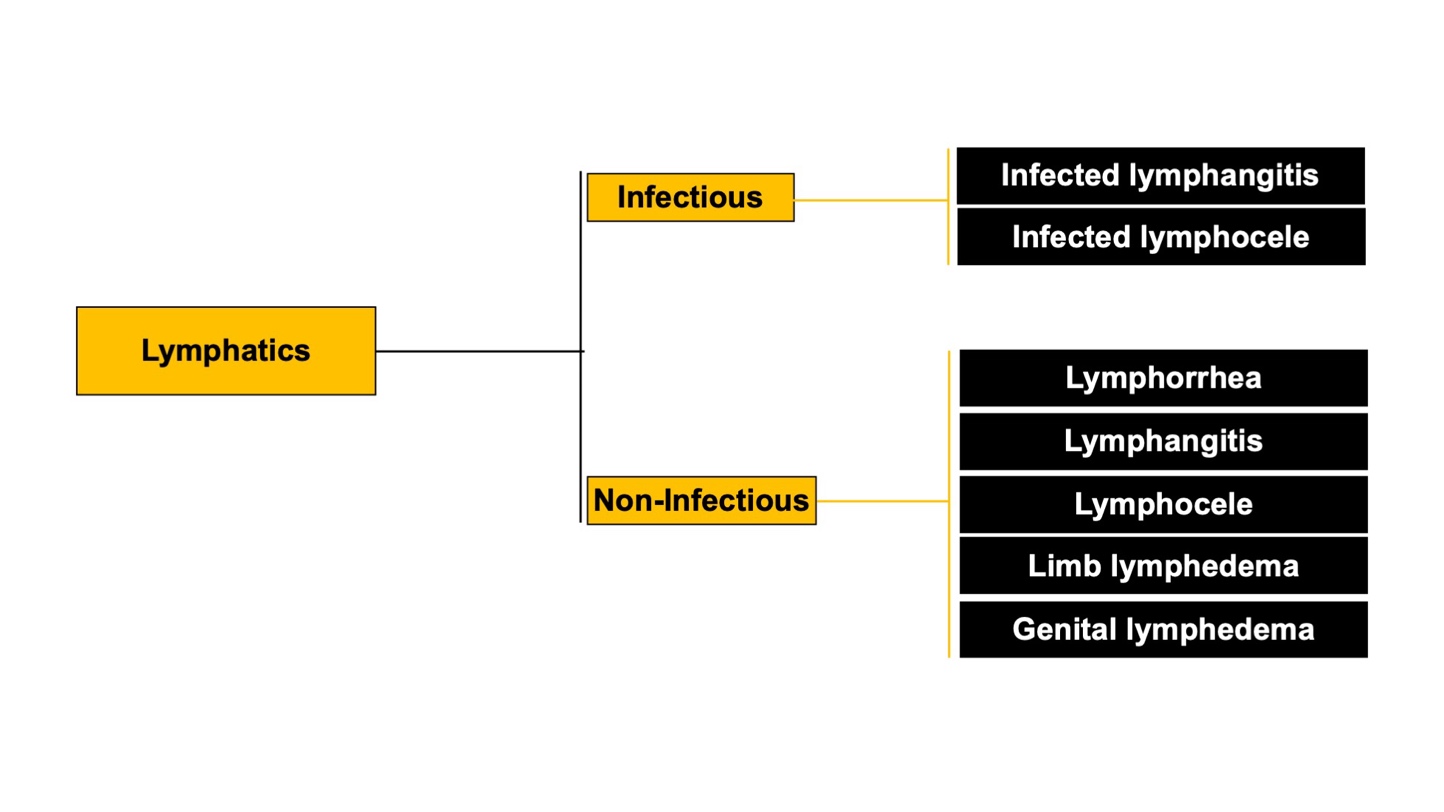

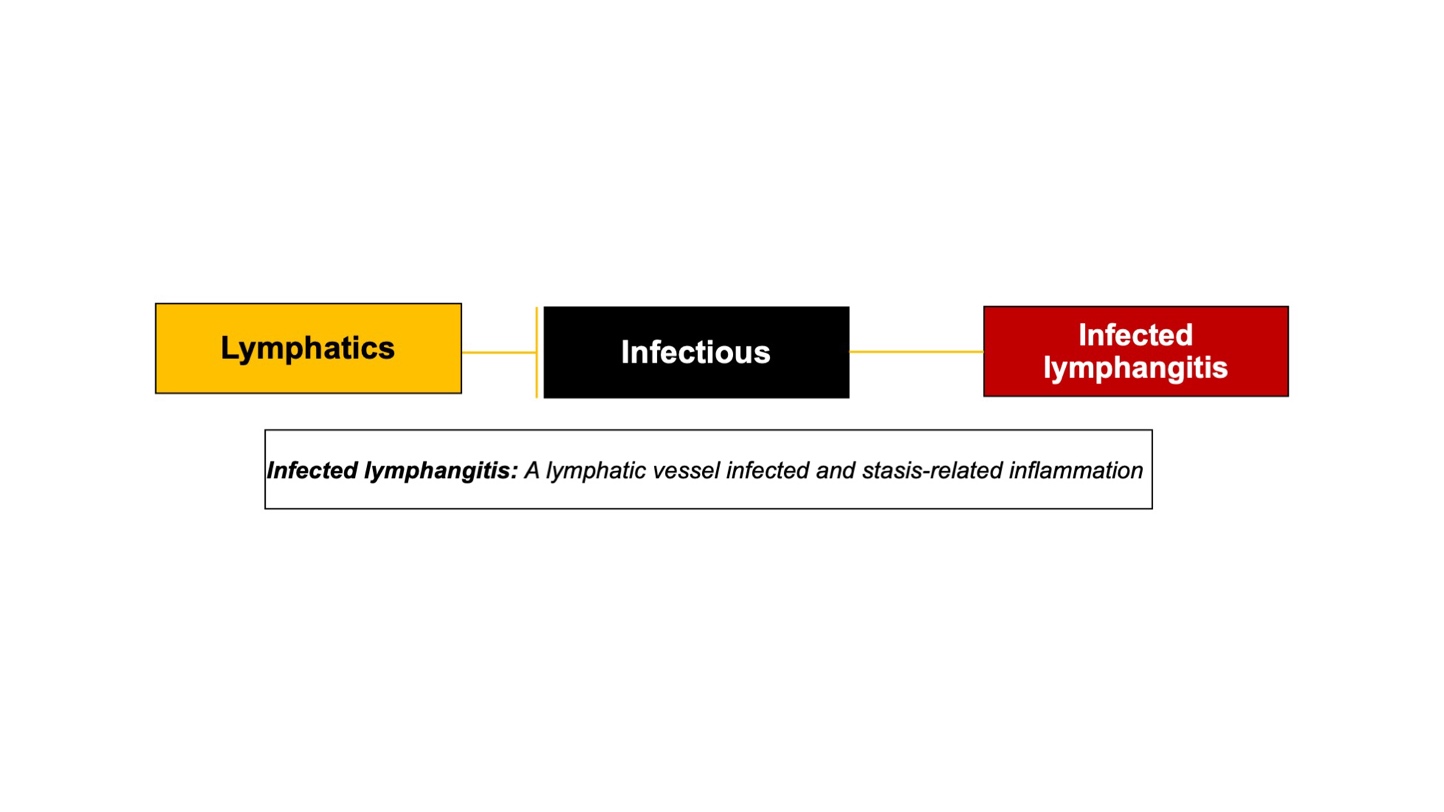

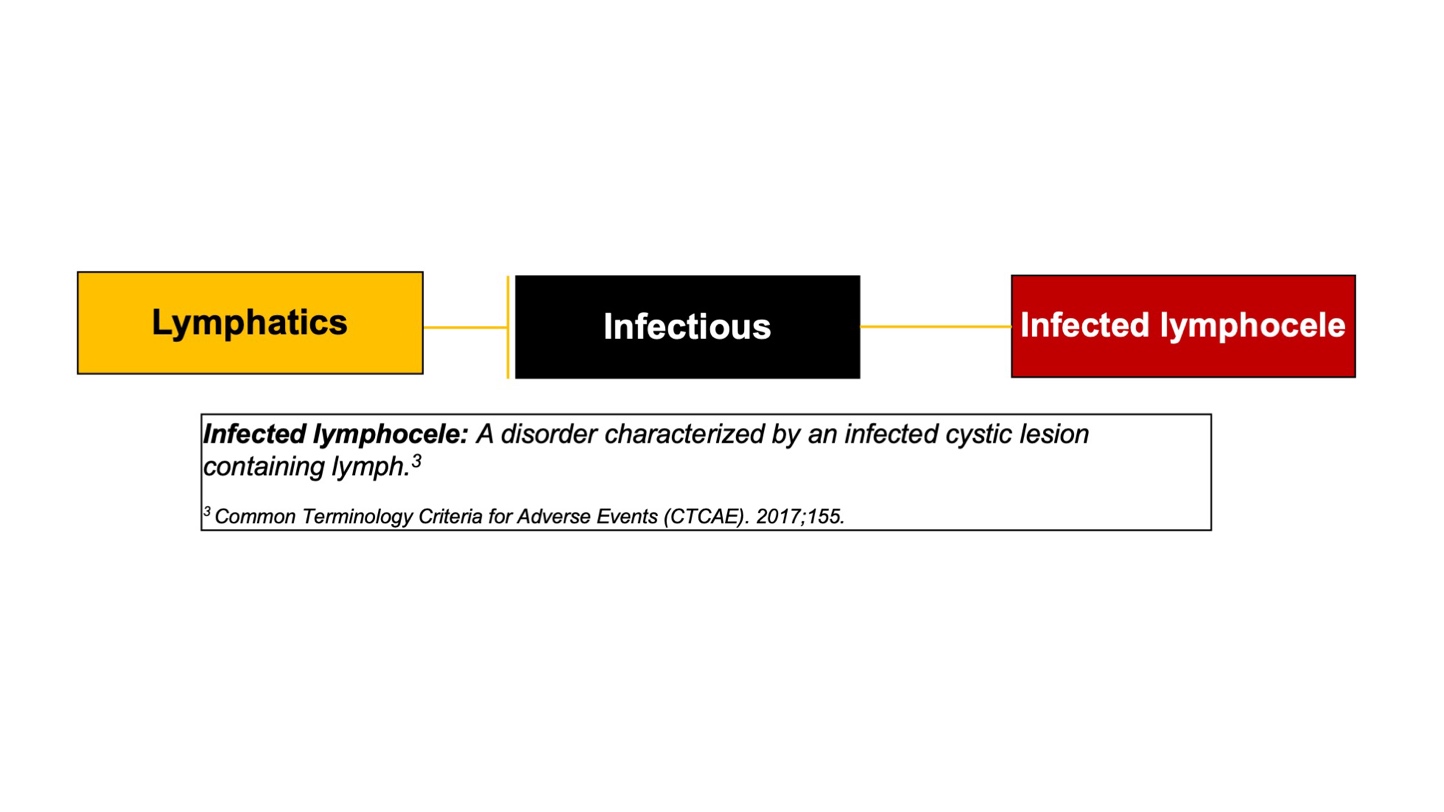

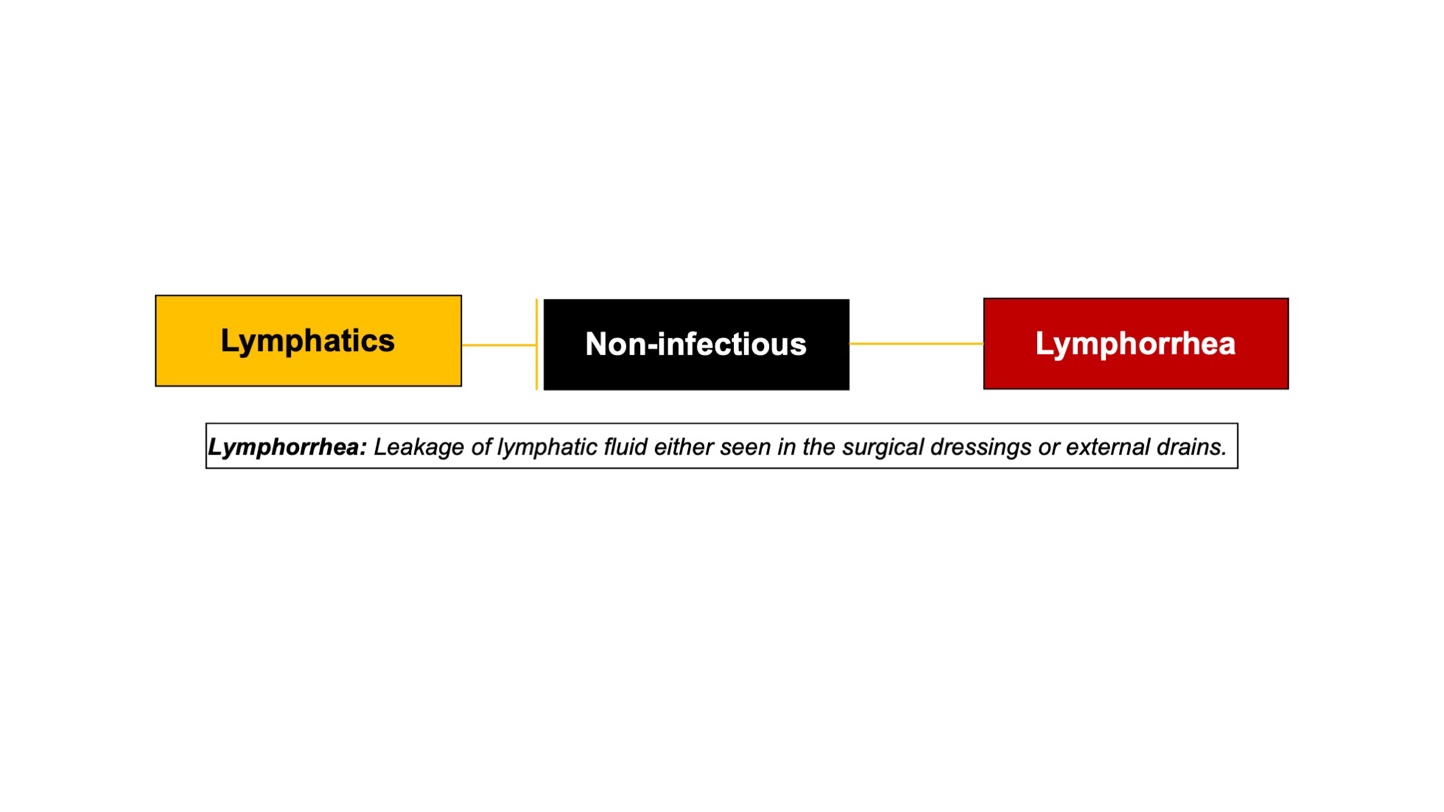

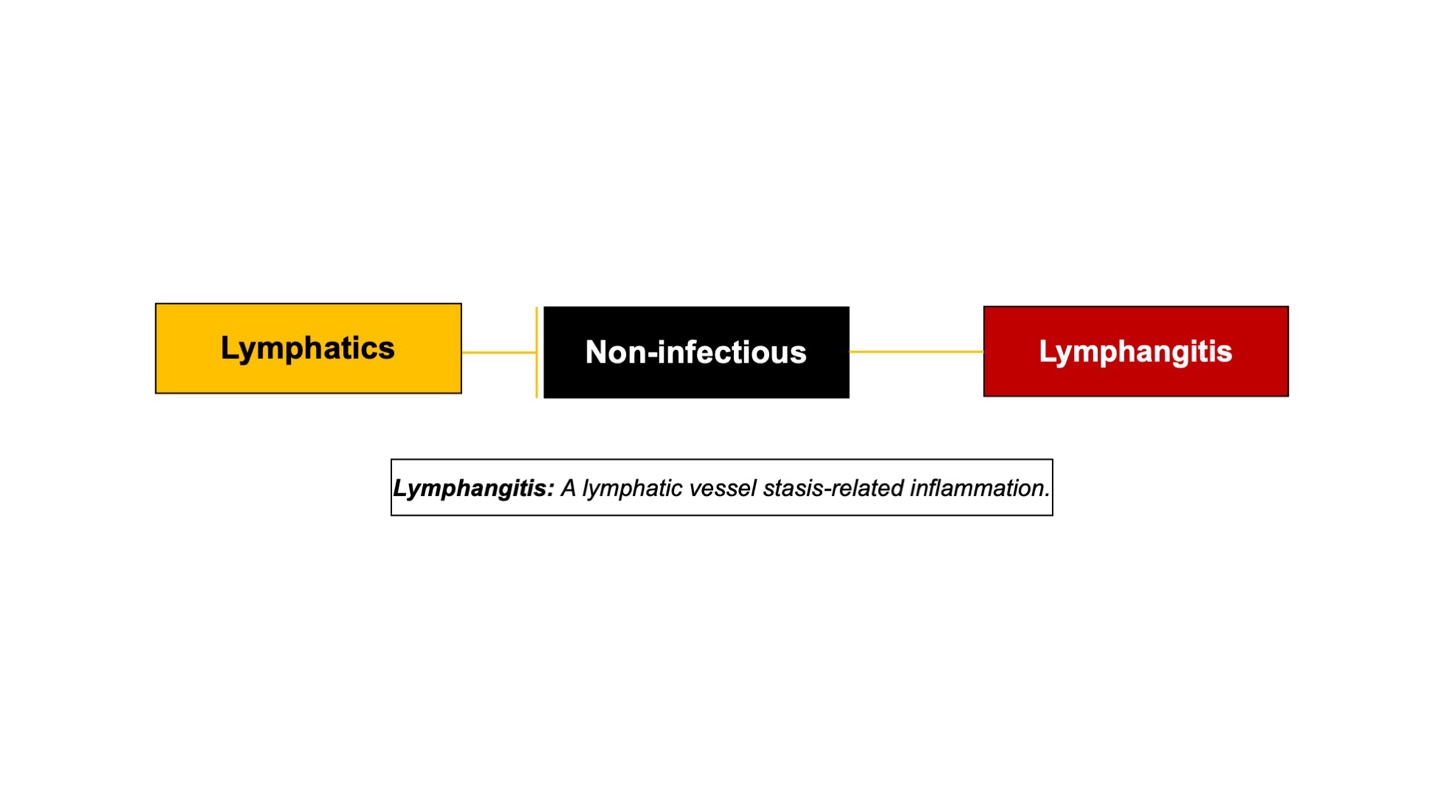

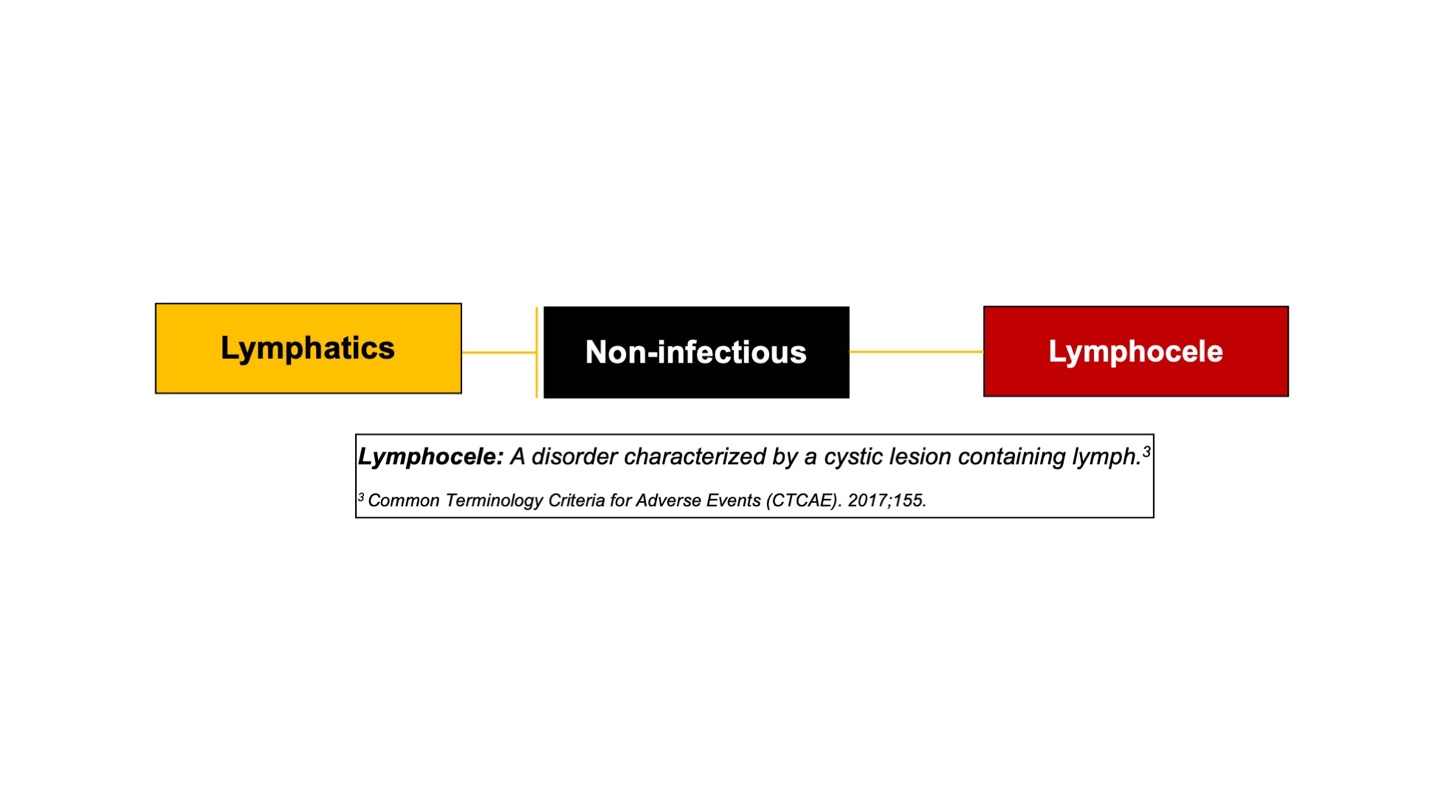

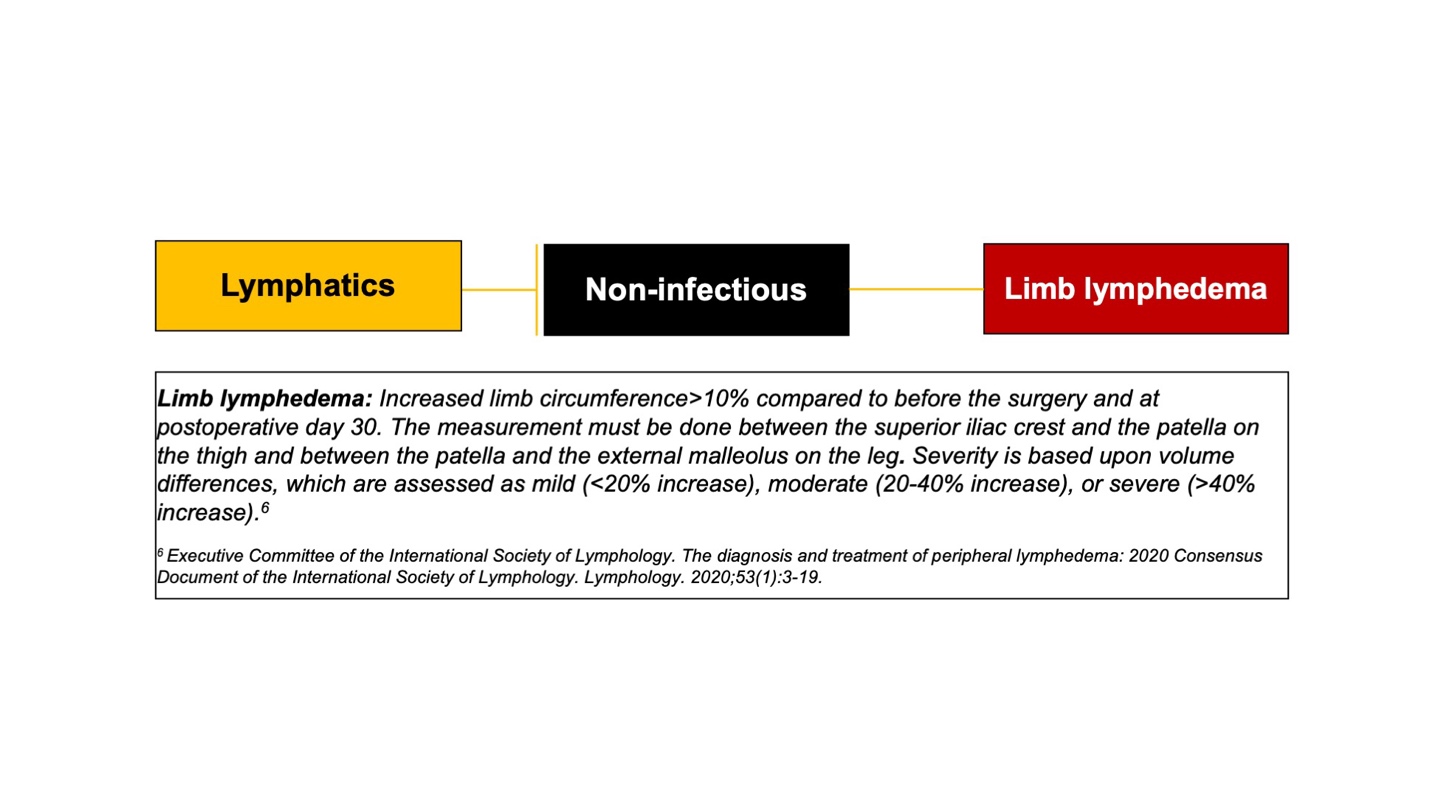

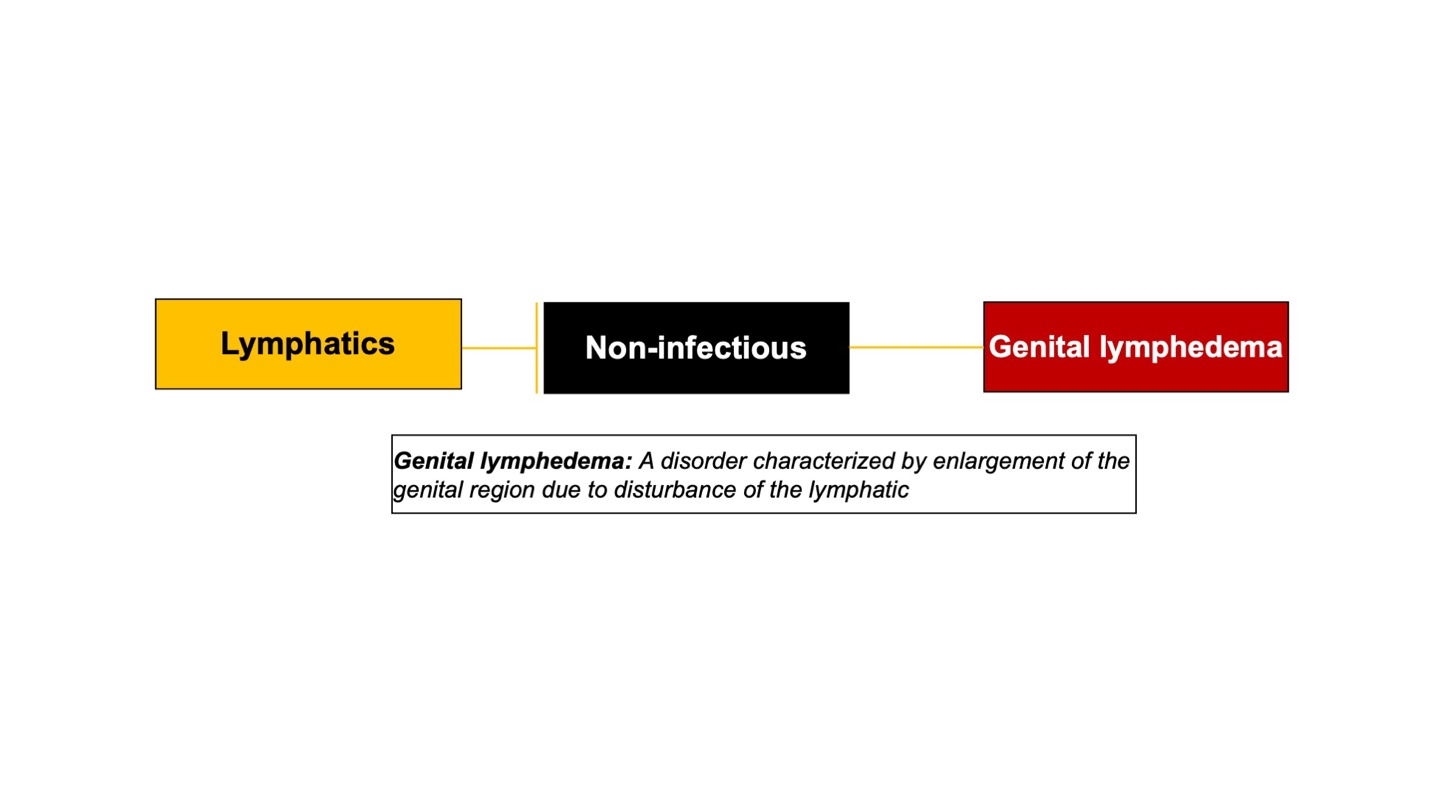

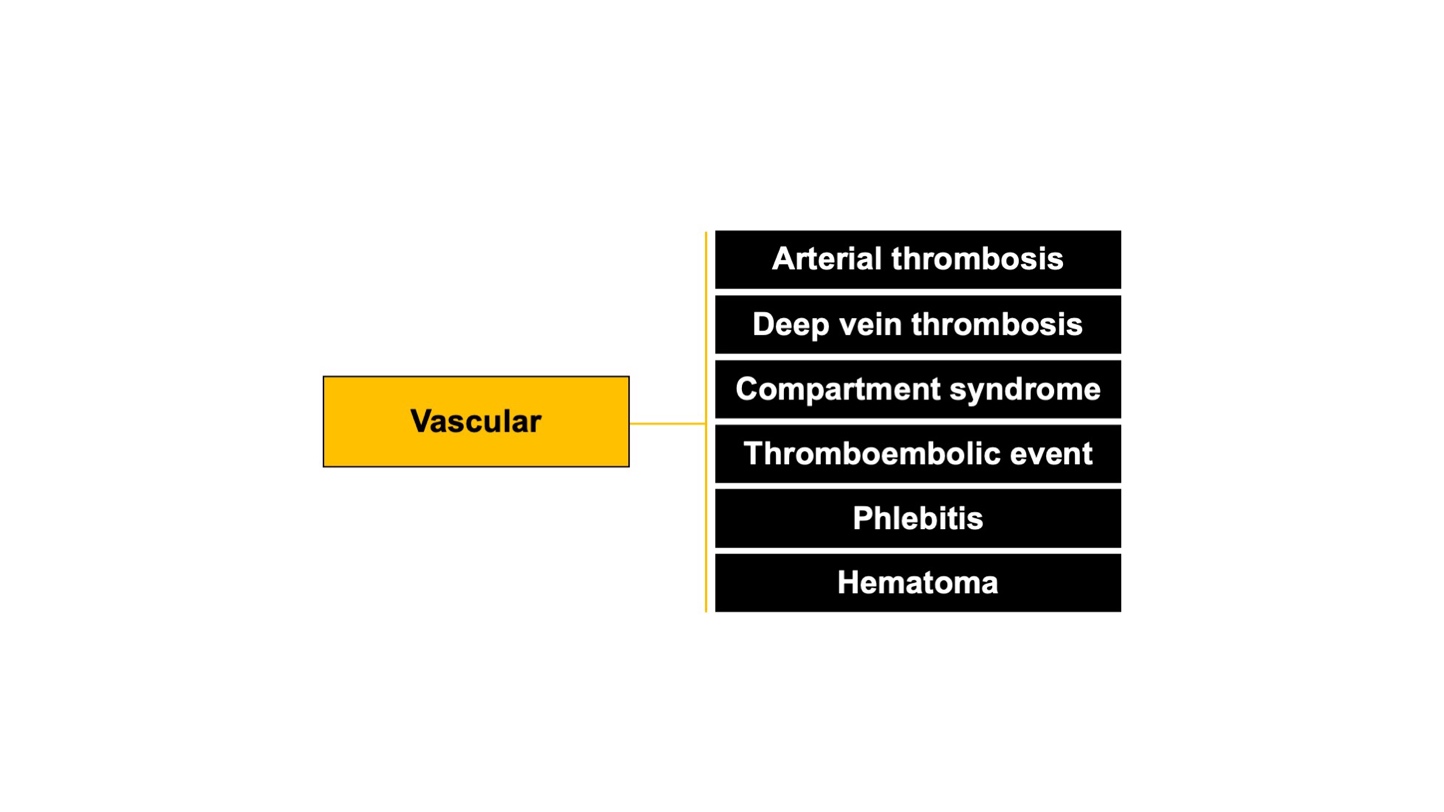

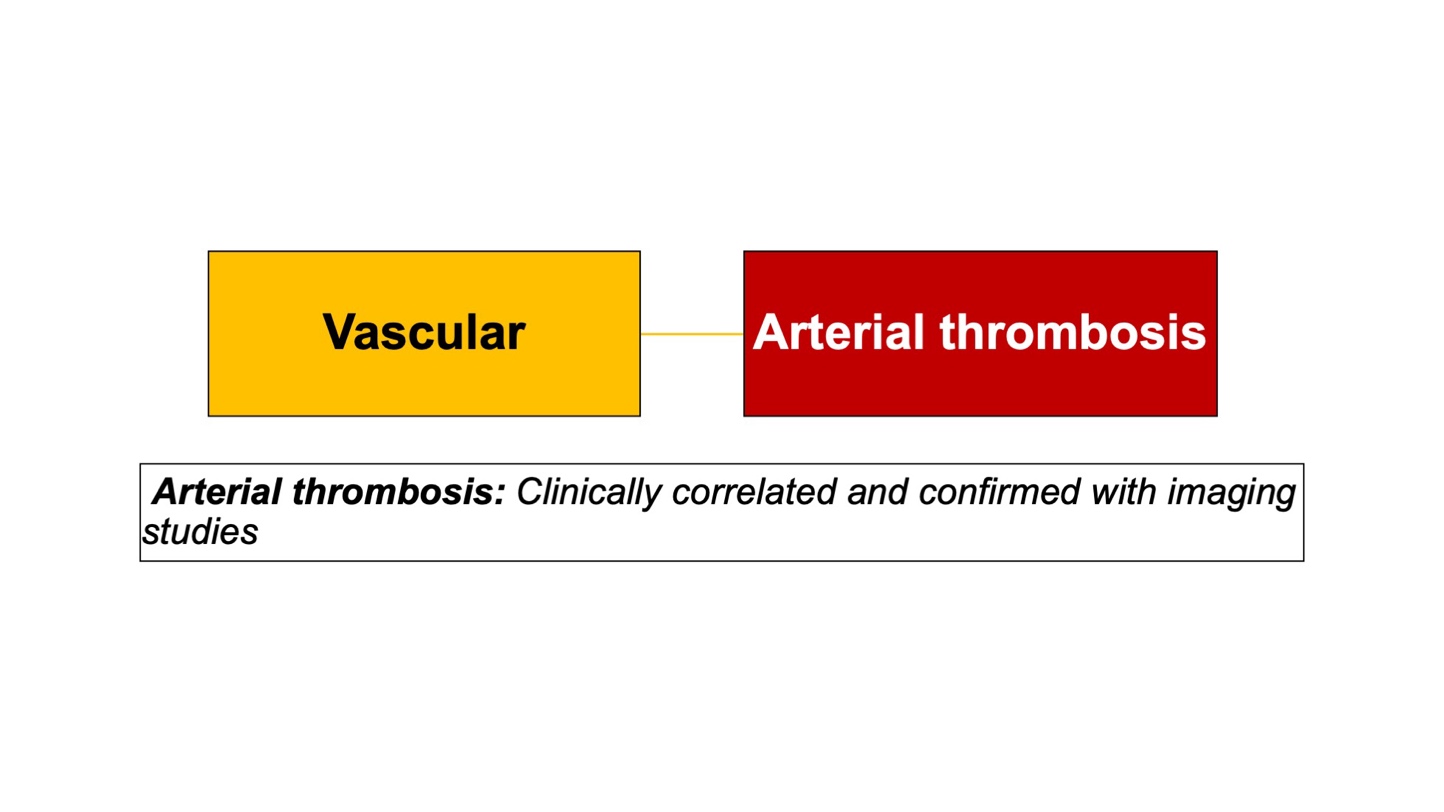

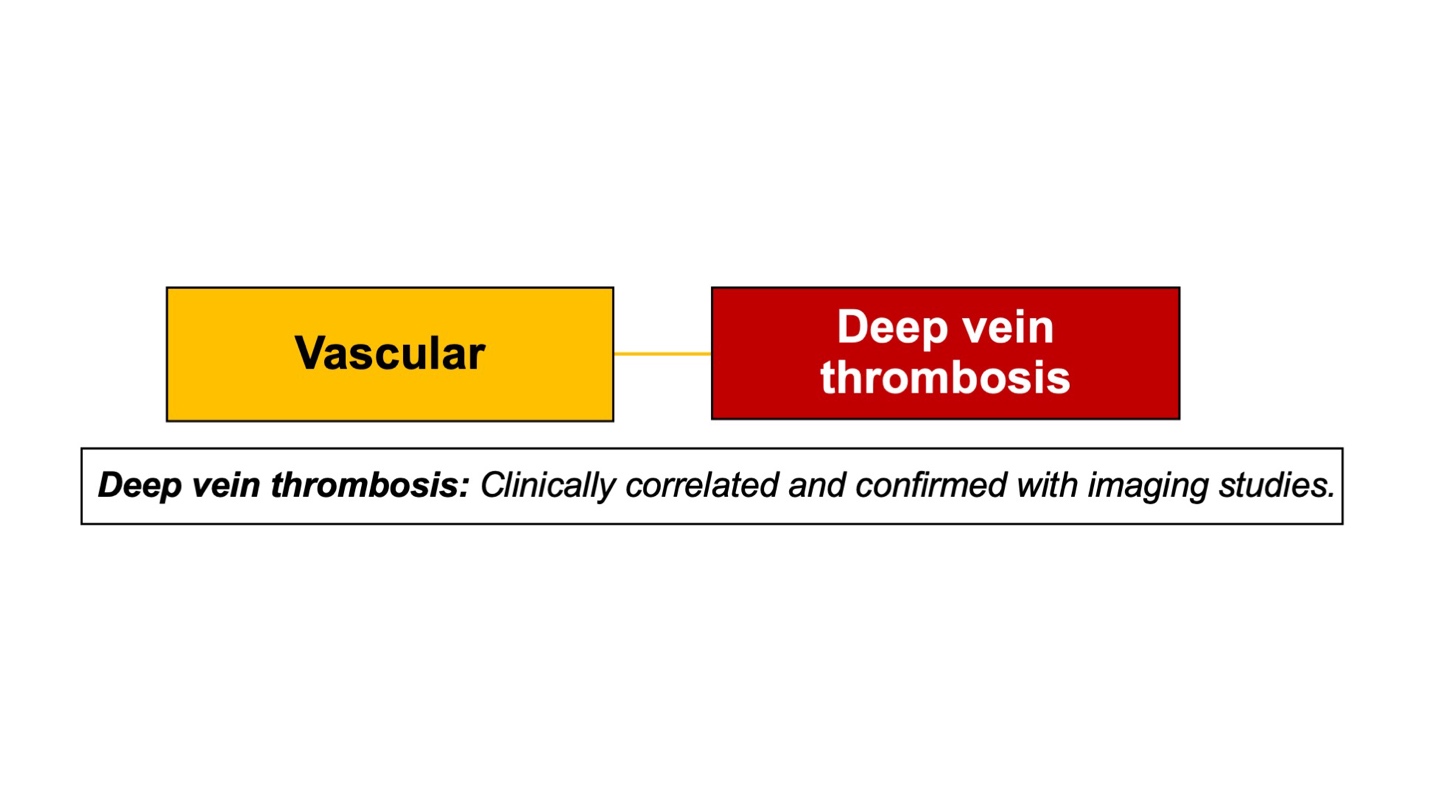

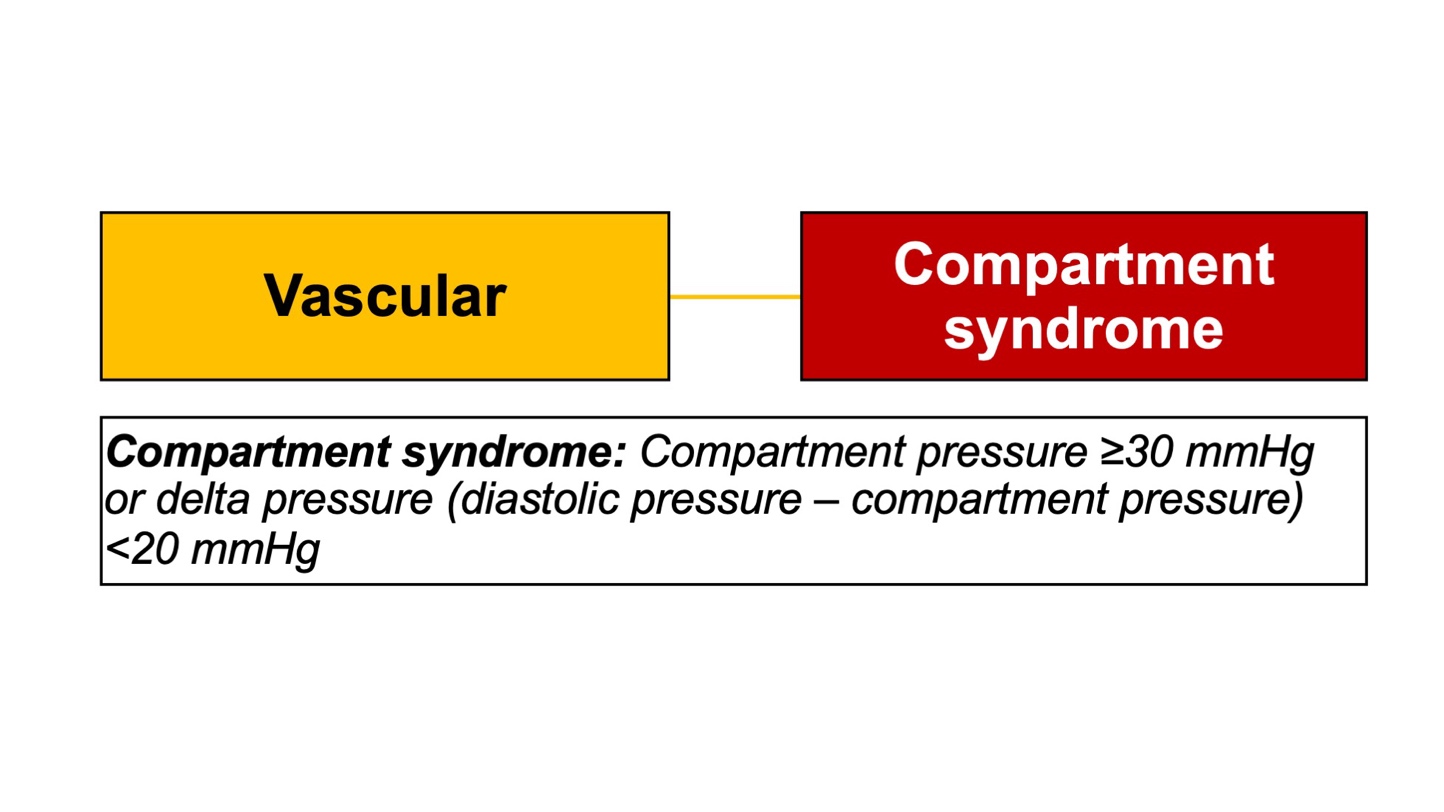

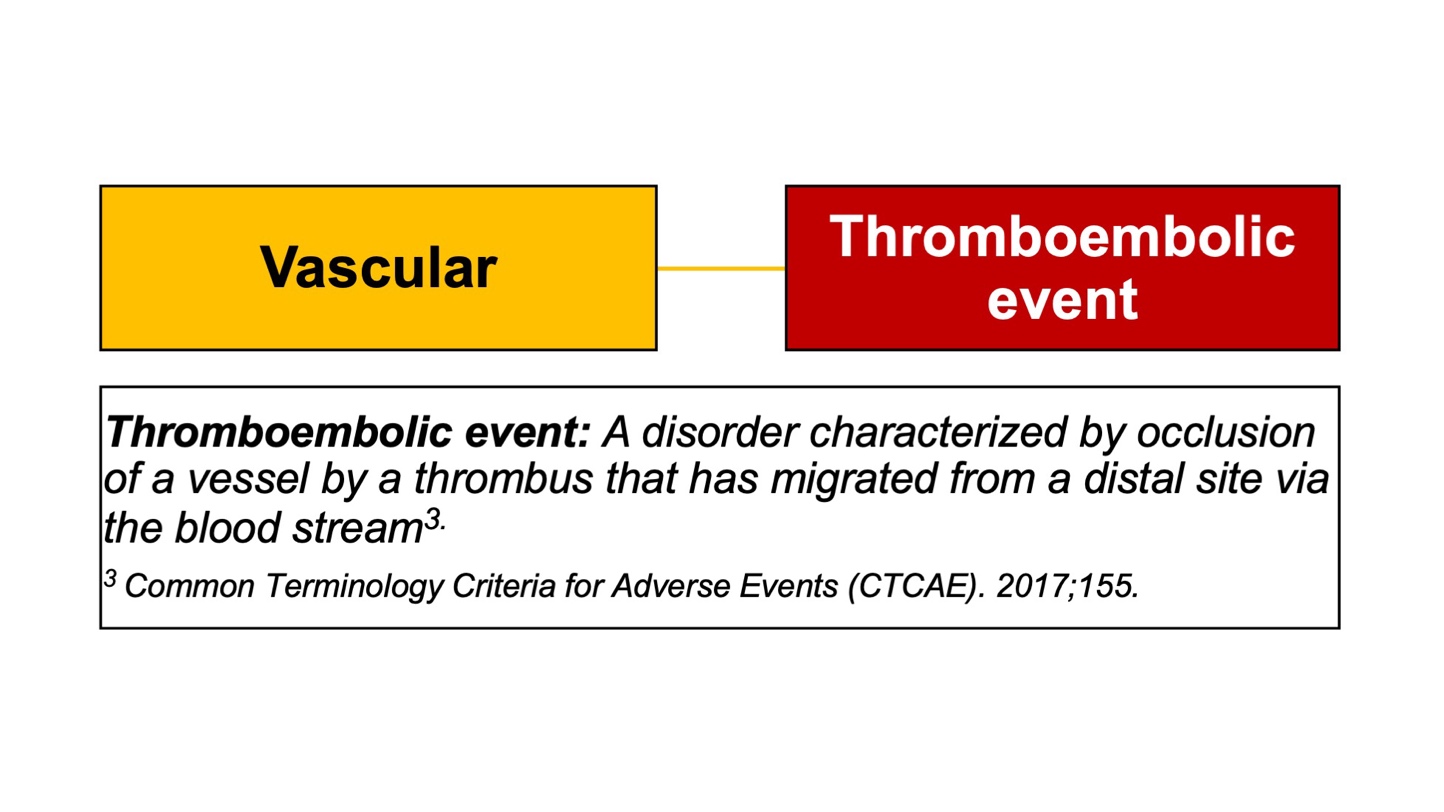

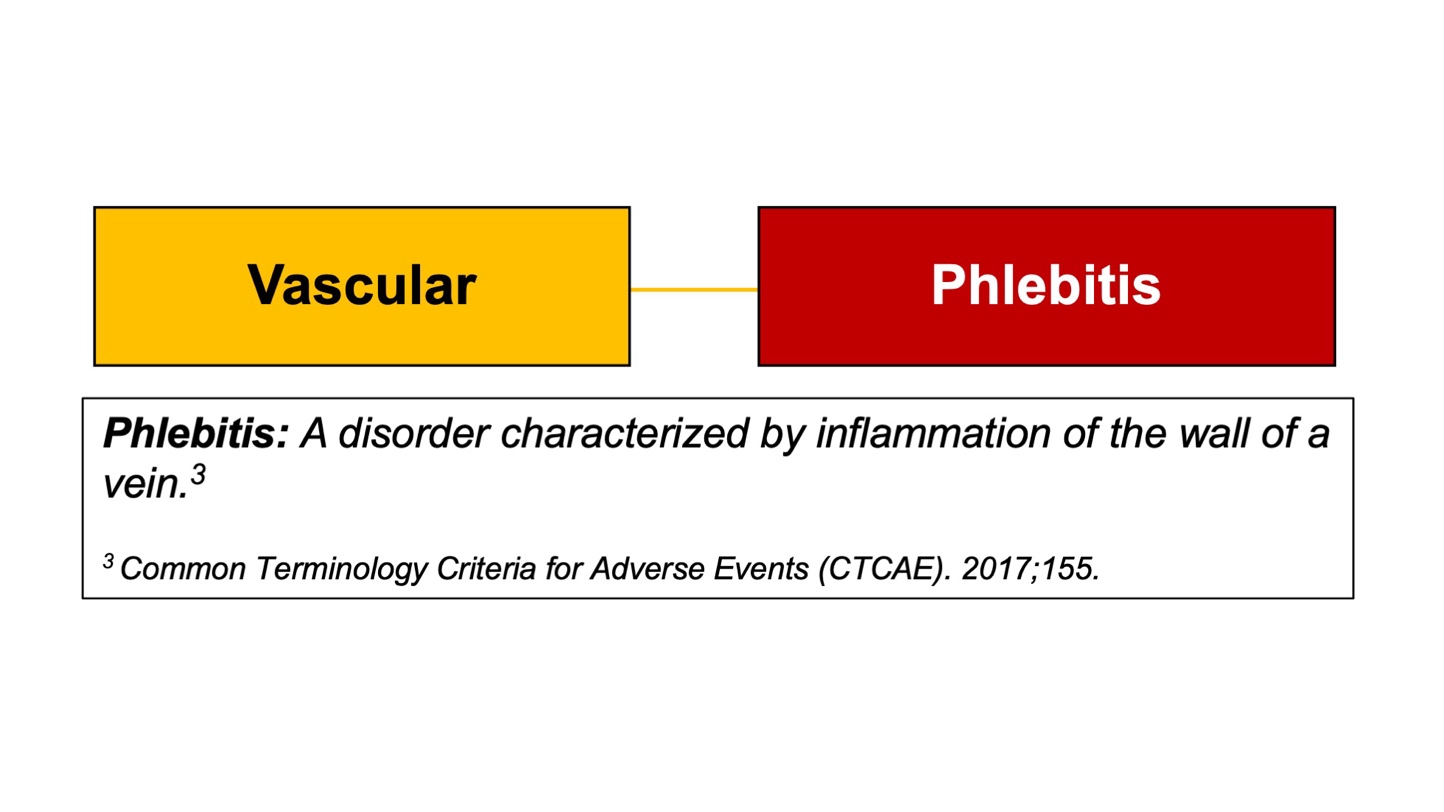

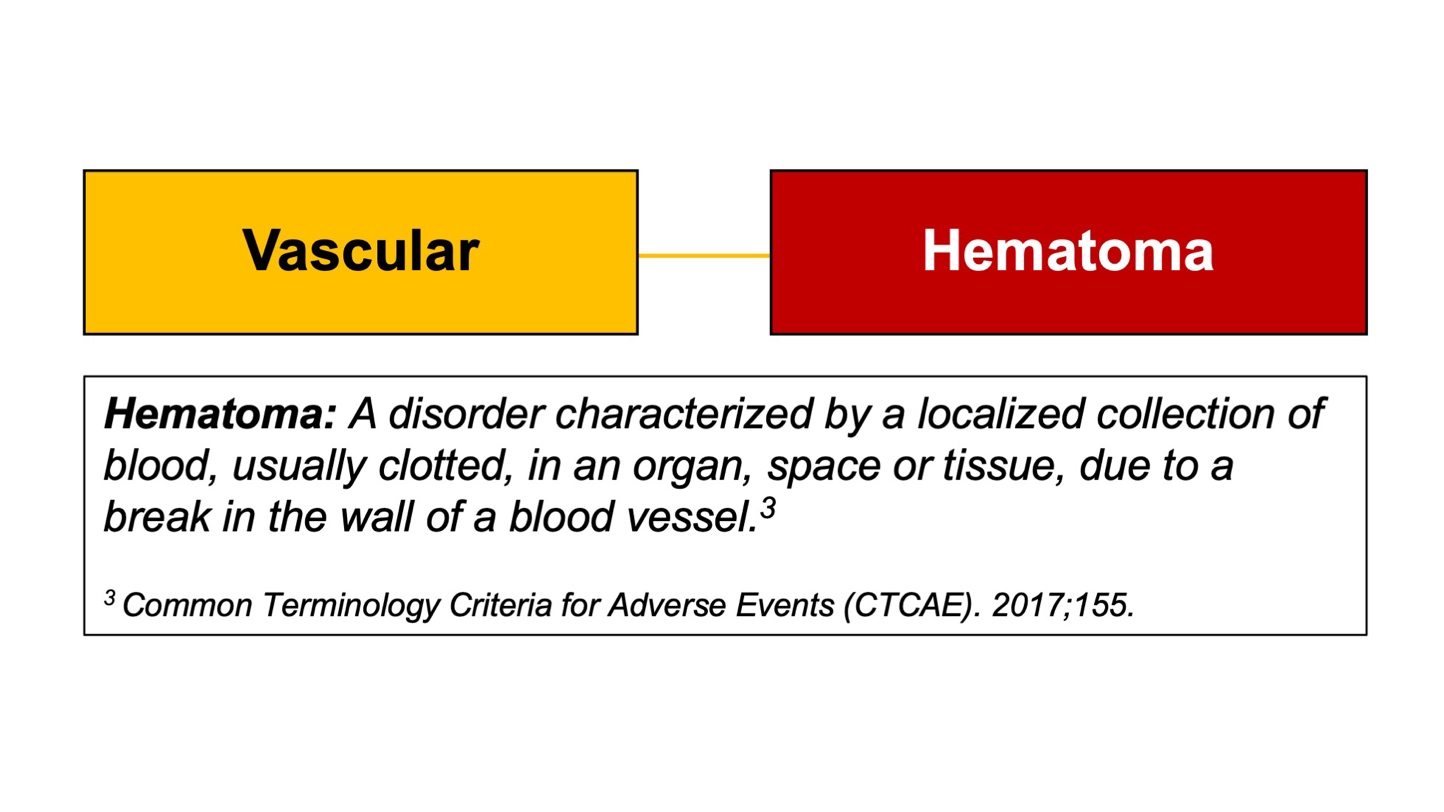

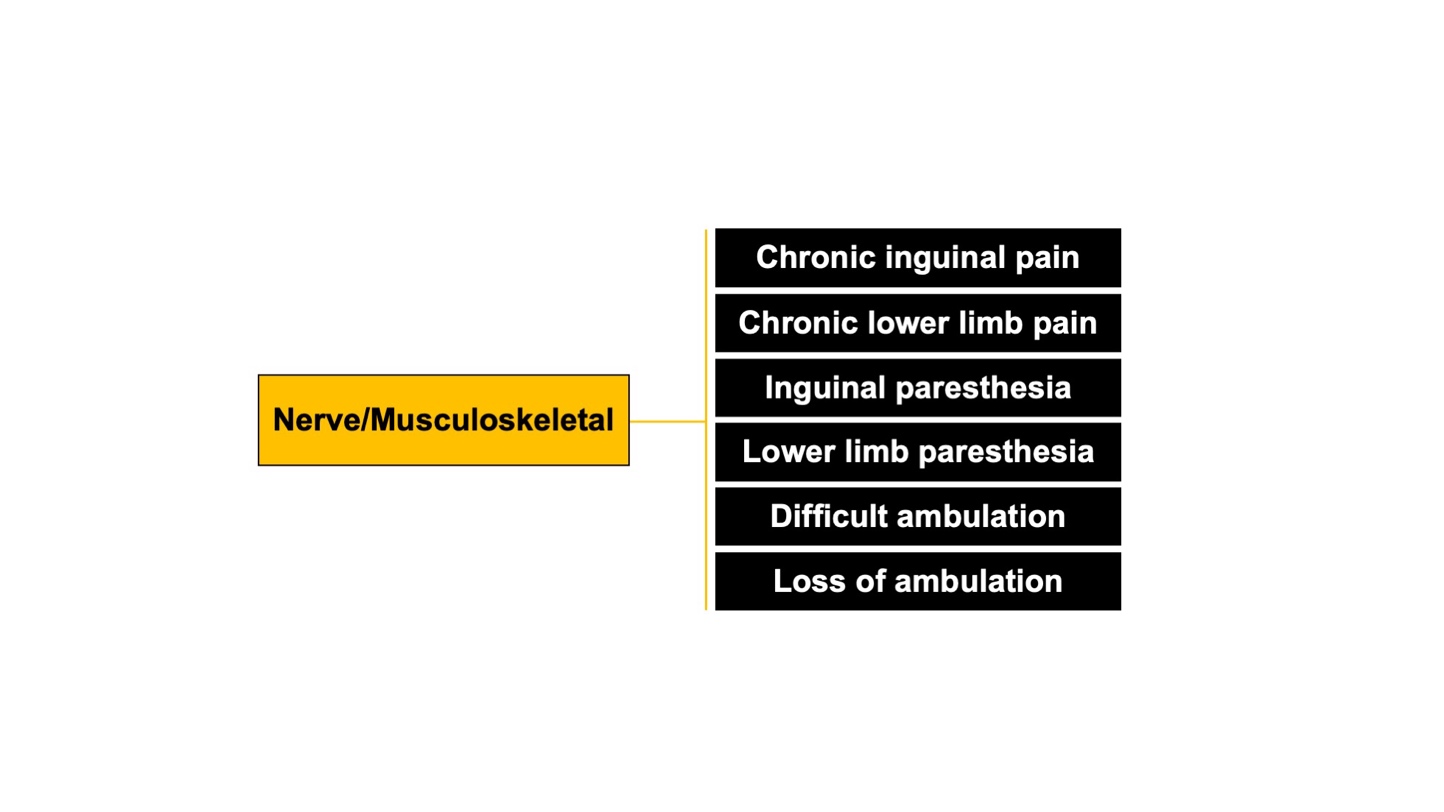

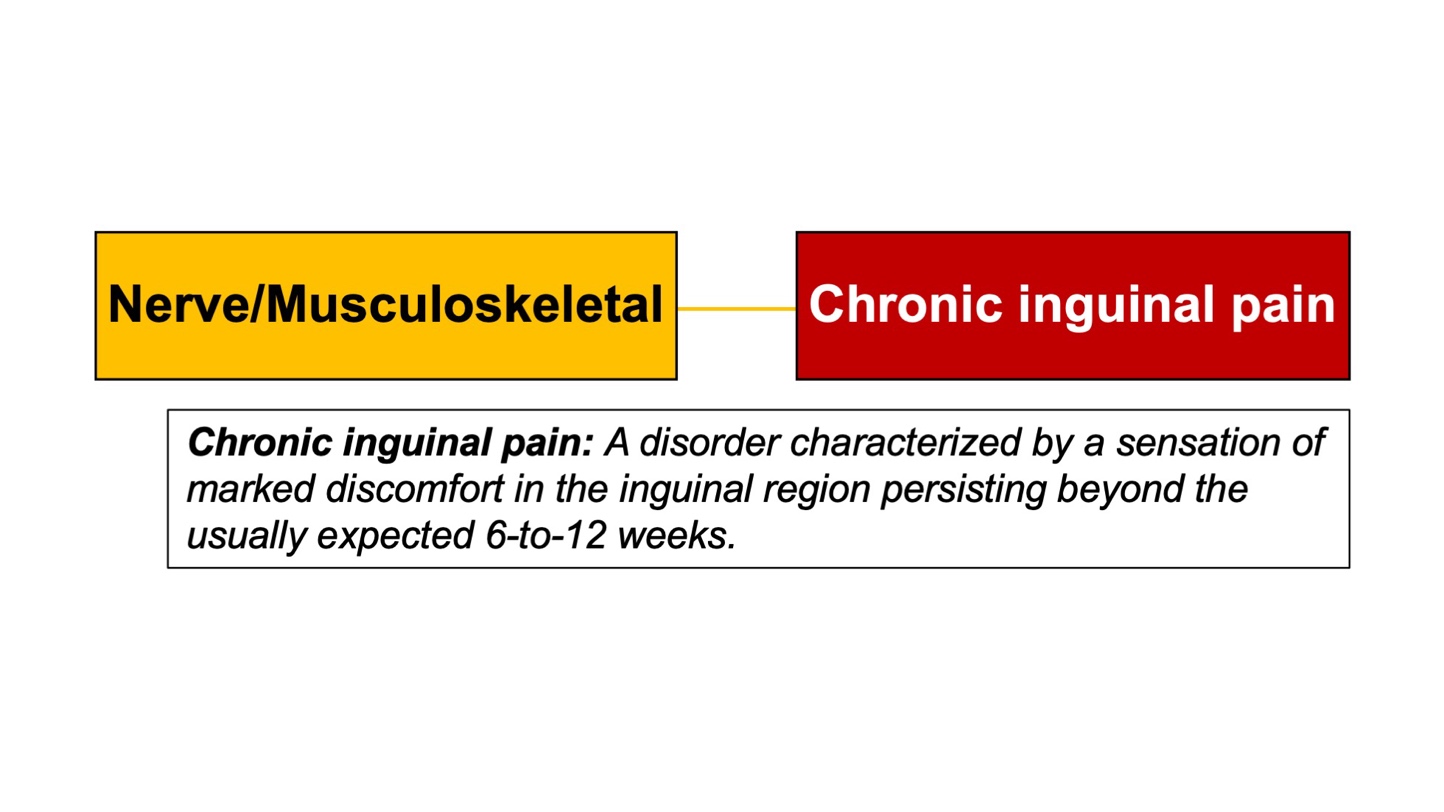

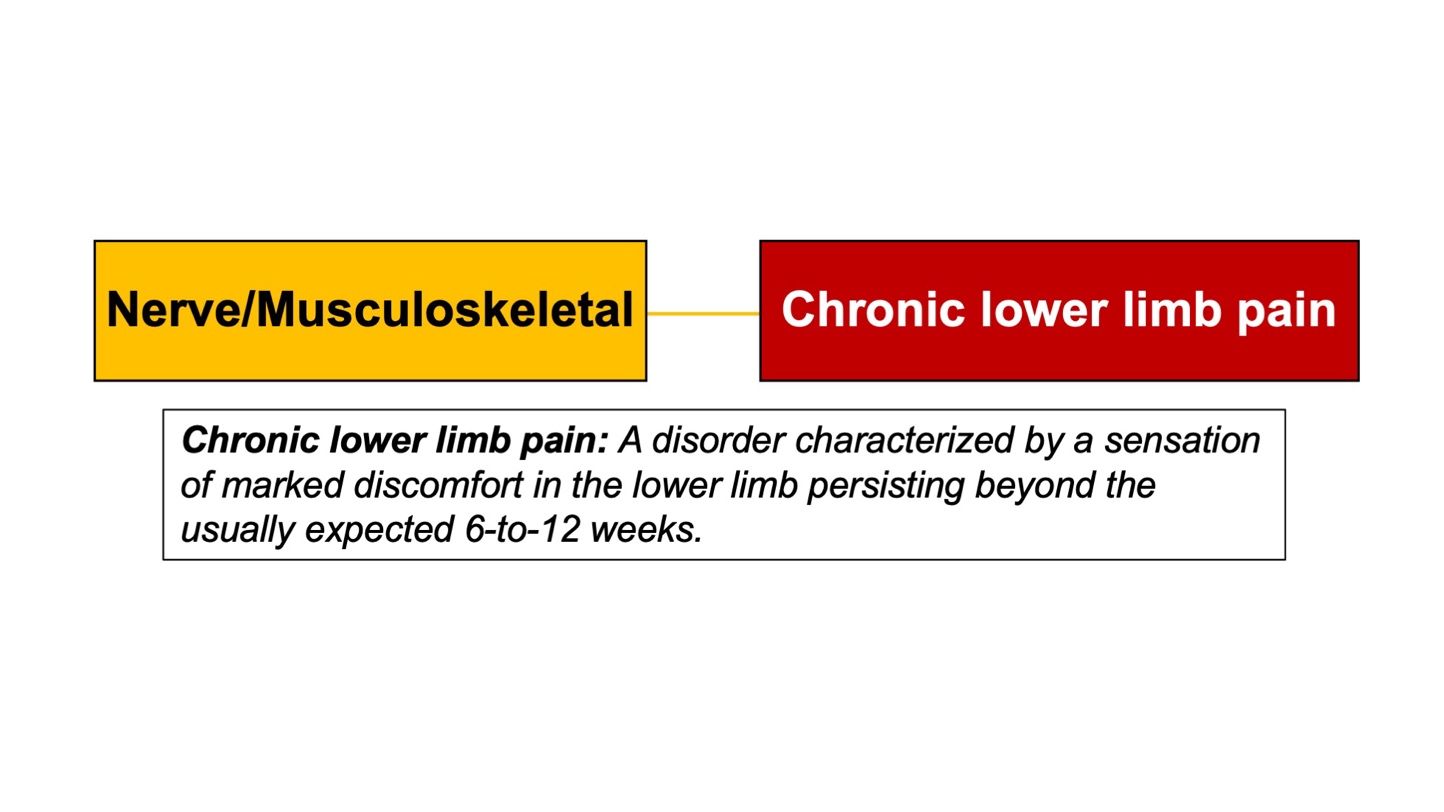

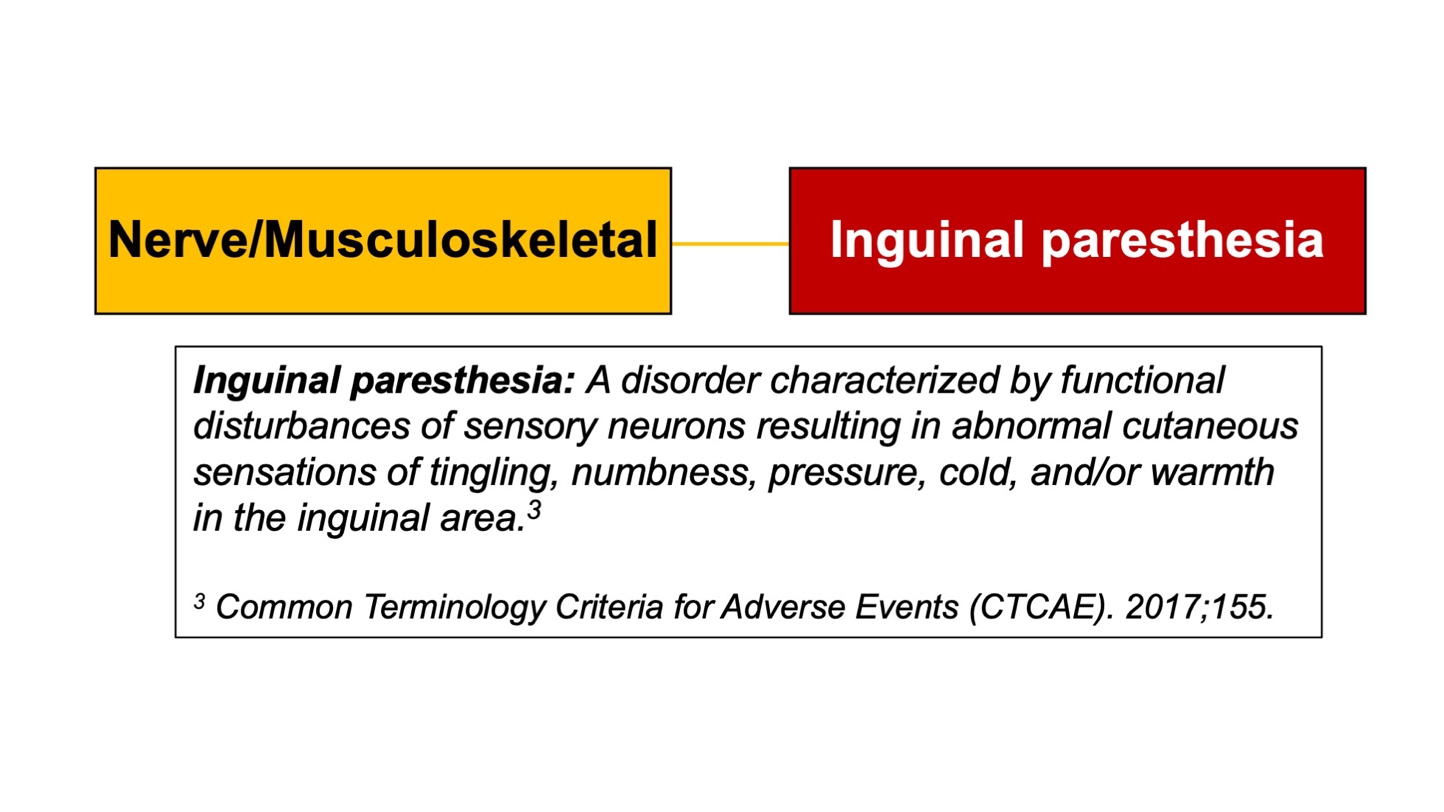

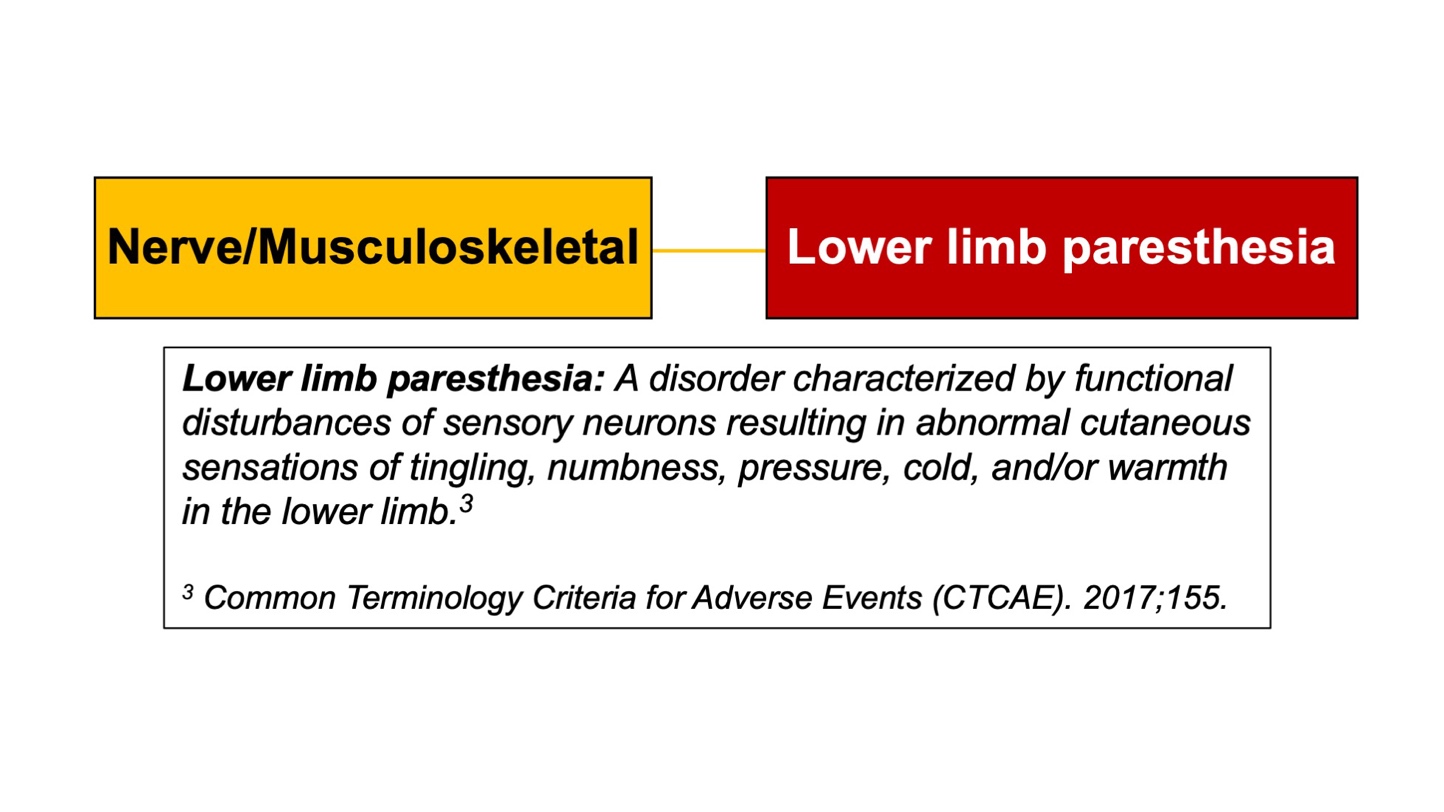

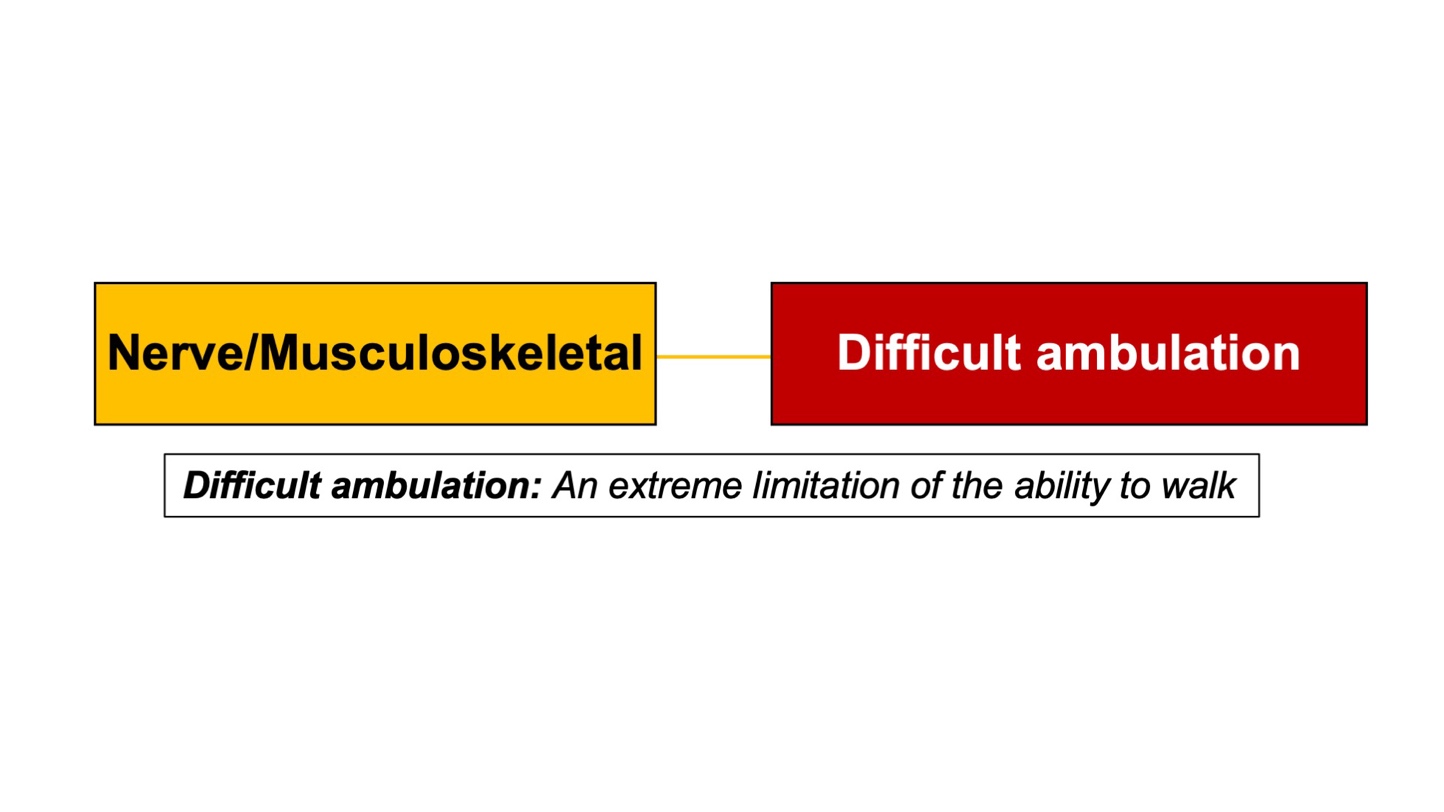

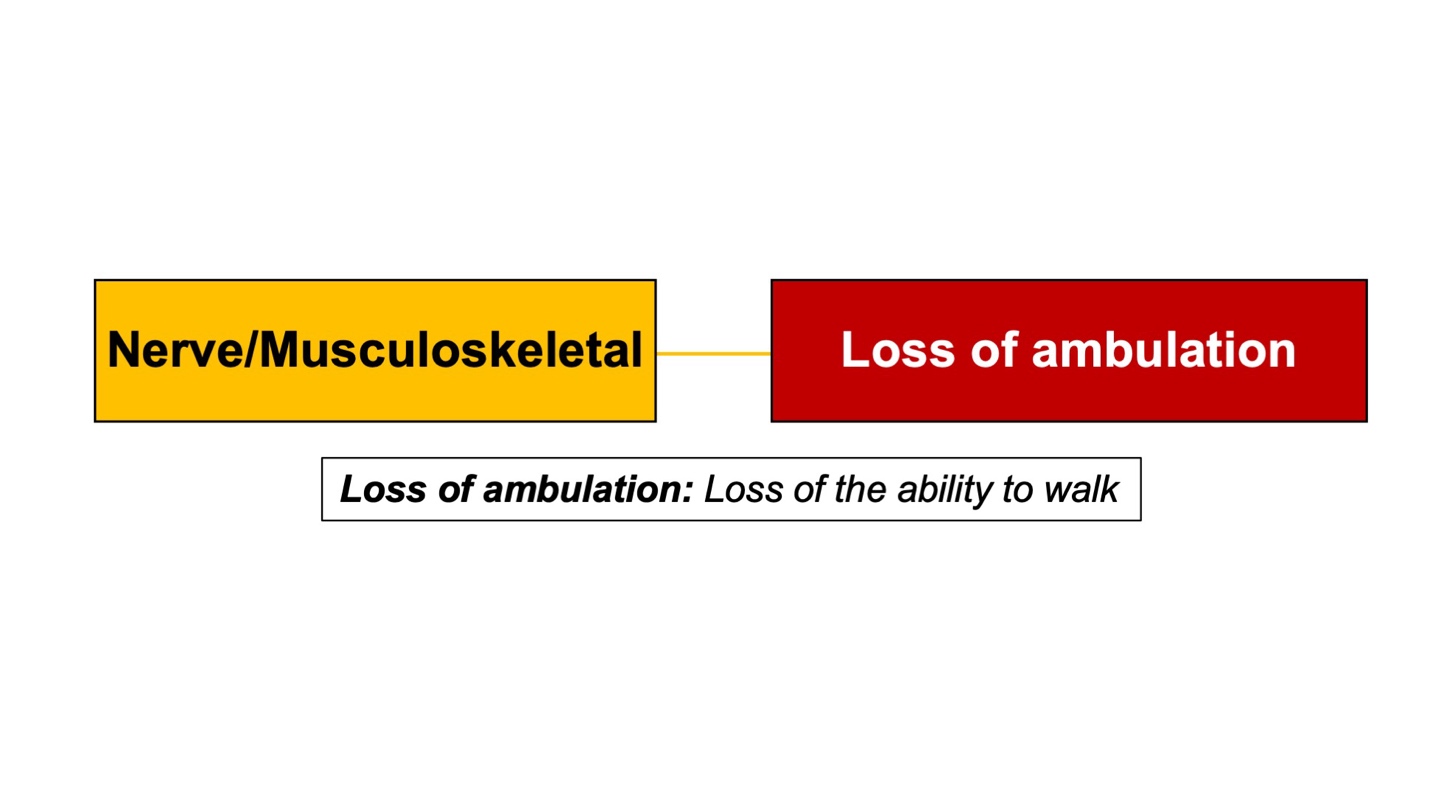
**

**
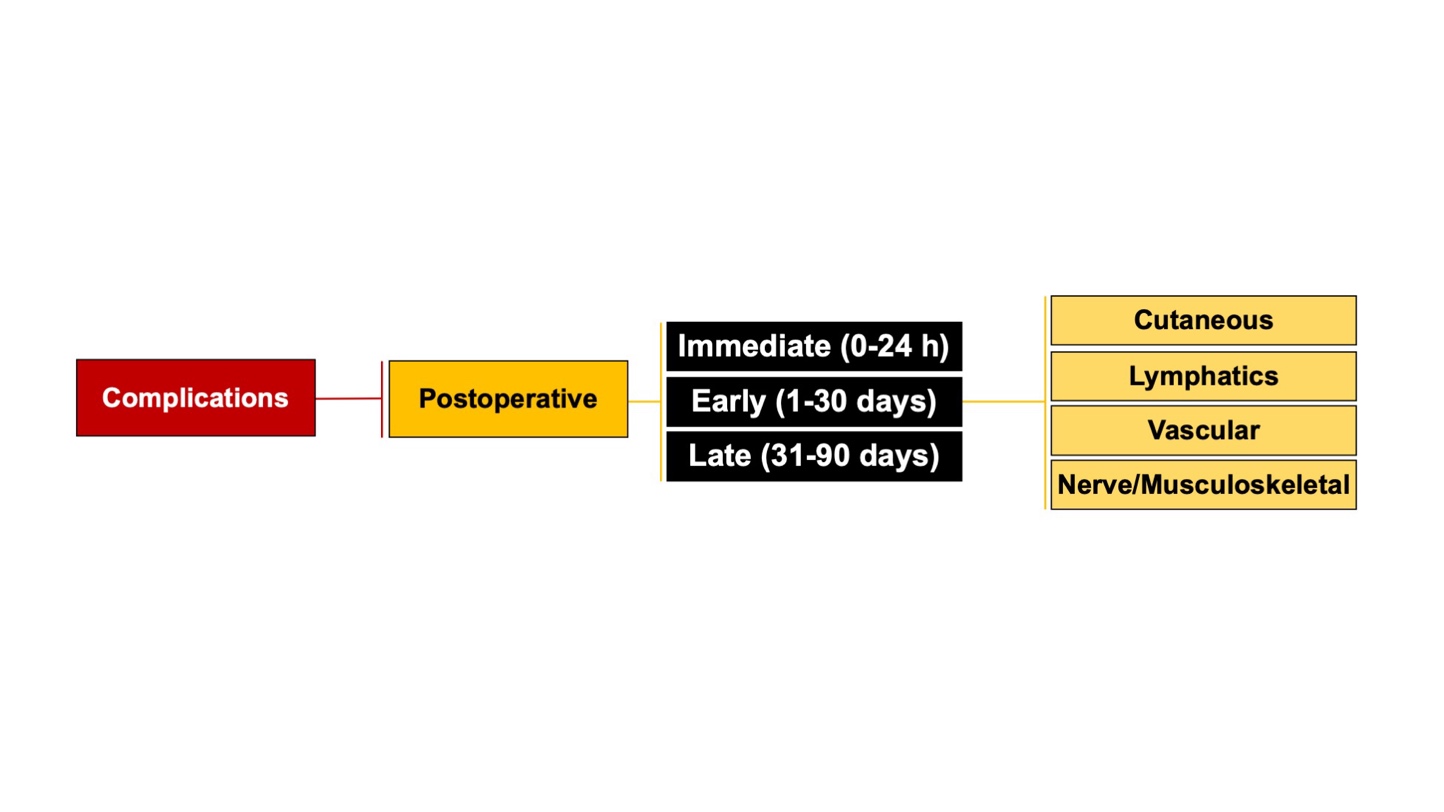
**

**
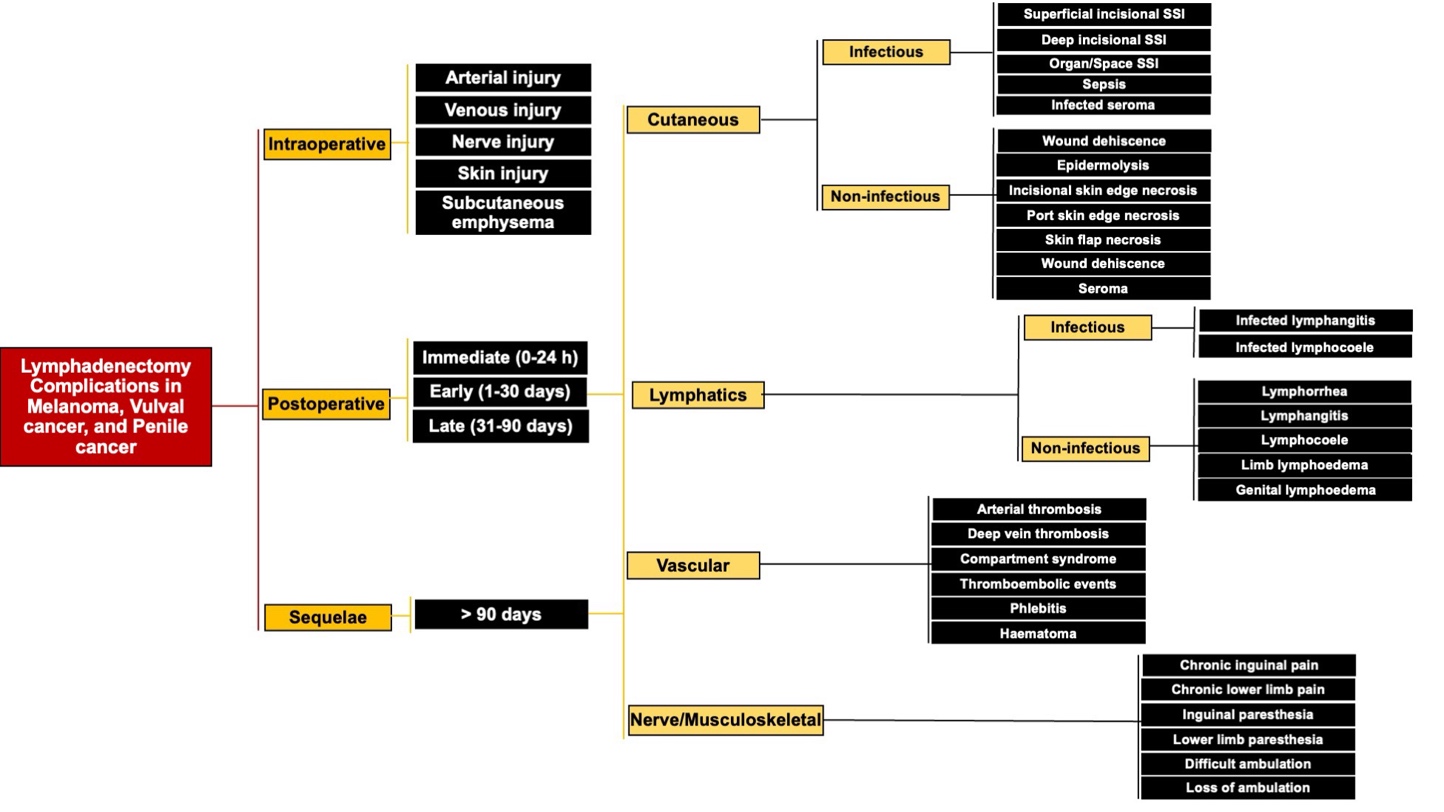
Supplementary Figures and Tables**

***Table 1.*** Amendments to the Delphi survey following the first round and panelists' feedback.

| Amendments to the Delphi survey following the first round and feedback | |
| --- | --- |
| # | **Amendments** |
| 1 | Inclusion of sequelae (>90 days) to the complications sub-classification. |
| 2 | Postoperative macro-categories were re-structured based on location/system rather than mixed etiology and site of complication that was proposed before.  The postoperative macro-categories proposed now are Cutaneous, Lymphatics, Vascular, and Nerve/Musculoskeletal. |
| 3 | The “infectious” complications were included and sub-classified within the cutaneous macro-category.  The cutaneous macro-category is sub-classified into infectious and non-infectious micro-categories |
| 4 | Improvement of “port skin edge necrosis” definition and inclusion into the cutaneous (non-infectious) category. |
| 5 | Improvement of “incisional skin edge necrosis” definition and inclusion into the cutaneous (non-infectious) category. |
| 6 | Improvement of lymphatics postoperative complications such as infected lymphocele included separately from lymphocele. |
| 7 | The term scrotal lymphedema was changed into genital lymphedema, which encloses vulvar cancer. |
| 8 | The hematoma complication was relocated from the cutaneous to the vascular macro-category. |
| 9 | The term pulmonary embolism was changed into thromboembolic events. |
| 10 | New CALI classification template based on previous suggestions/amendments. |

| Amendments to the Delphi survey following the second round and feedback | |
| --- | --- |
| # | **Amendments** |
| 1 | Consider “hypercapnia” as an anesthesia-related complication and not related to ILND. Therefore, exclude from the classification. |
| 2 | Include “seroma” within the cutaneous (non-infectious) category, and separately “infected seroma” within the cutaneous (infectious) category. |
| 3 | Re-assess if “epidermolysis” should be defined, classified, and included within the cutaneous (non-infectious) category. |
| 4 | The “lymphatics” complications were sub-classified into infectious and non-infectious with their respective complications included |
| 5 | Final CALI classification template based on previous suggestions/amendments. |

***Table 2.*** Amendments to the Delphi survey following the second round and panelists' feedback.
